# Thalamic circuits for independent control of prefrontal signal and noise

**DOI:** 10.1038/s41586-021-04056-3

**Published:** 2021-10-06

**Authors:** Arghya Mukherjee, Norman H. Lam, Ralf D. Wimmer, Michael M. Halassa

**Affiliations:** 1 McGovern Institute for Brain Research, Massachusetts Institute of Technology, Cambridge, MA USA; 2 Department of Brain and Cognitive Sciences, Massachusetts Institute of Technology, Cambridge, MA USA

**Keywords:** Cognitive control, Neurophysiology

## Abstract

Interactions between the mediodorsal thalamus and the prefrontal cortex are critical for cognition. Studies in humans indicate that these interactions may resolve uncertainty in decision-making^1^, but the precise mechanisms are unknown. Here we identify two distinct mediodorsal projections to the prefrontal cortex that have complementary mechanistic roles in decision-making under uncertainty. Specifically, we found that a dopamine receptor (D2)-expressing projection amplifies prefrontal signals when task inputs are sparse and a kainate receptor (GRIK4) expressing-projection suppresses prefrontal noise when task inputs are dense but conflicting. Collectively, our data suggest that there are distinct brain mechanisms for handling uncertainty due to low signals versus uncertainty due to high noise, and provide a mechanistic entry point for correcting decision-making abnormalities in disorders that have a prominent prefrontal component^2–6^.

## Abstract (web summary)

Two different cell types in the mediodorsal thalamus have complementary roles in decision-making, with one type of mediodorsal projection amplifying prefrontal activity under low signal levels and one type suppressing it under high noise levels.

## Main

**Fig. 1:**
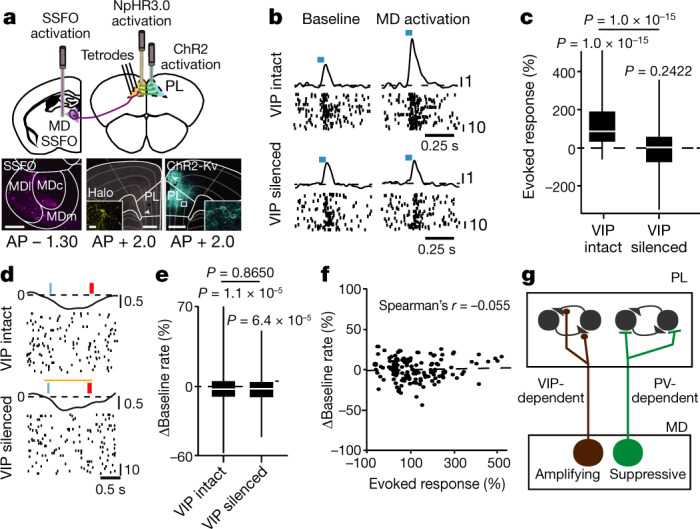
MD amplifies functional PFC connectivity through cortical VIP^+^ interneurons. **a**, Top, cartoon of experimental set-up. Bottom left, stabilized step function opsin (SSFO) MD expression. Bottom middle, PL tetrode location (white arrow); eNHpR3.0-expressing VIP^+^ neuron (inset). Bottom right, somatic ChR2 in contralateral PL. MDl, lateral MD; MDc, central MD; MDm, medial MD. Scale bars, 200 μm; 20 μm (inset). **b**, Top, putative excitatory PL neuron showing amplification of its response to intracortical stimulation (blue tick) when the MD is activated. Bottom, this effect is eliminated by inactivation of local VIP^+^ interneurons. **c**, Population quantification of effect in **b** (*n* = 151 excitatory PL neurons). **d**, Baseline spike rate suppression in another PL neuron after MD activation is unaffected by VIP^+^ interneuron inactivation. **e**, Population quantification of effect in **d** (*n* = 373 neurons). **f**, The two MD effects are uncorrelated (*n* = 151 neurons). **g**, Hypothesized MD projections target prefrontal interneurons for independent control over amplification and suppression of cortical activity patterns. Data from 4 VIP-cre mice. For **c**, **e** Mann-Whitney *U* for comparisons to baseline; Wilcoxon signed-rank for group comparisons. All statistical tests are two-tailed. For box plots in **c**, **e**, boundaries, 25–75th percentiles; midline, median; whiskers, minimum–maximum. Source data.

**Extended Data Fig. 1:**
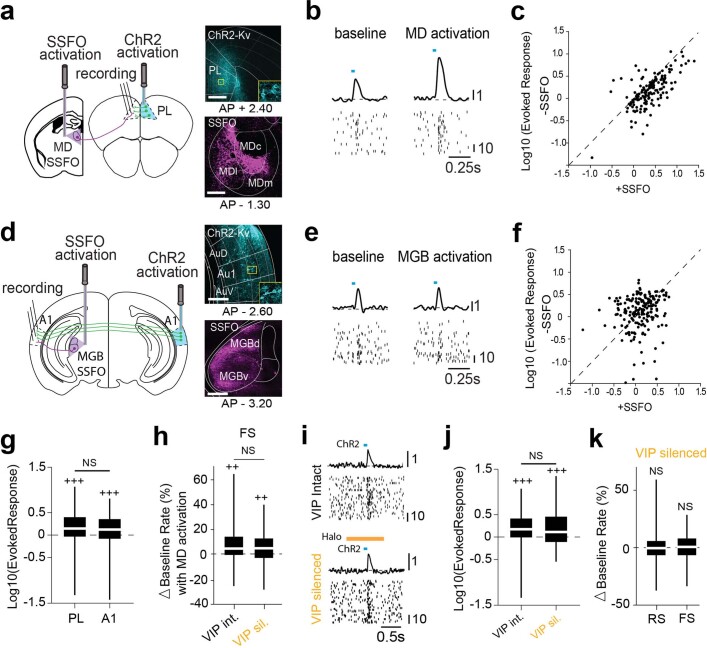
Distinct effect of MD activation on PL activity compared to MGB on A1 and controls relevant to VIP^+^ mediation of MD-driven amplification of PL connectivity. **a**, Left: Cartoon of setup testing the role of MD thalamus activation on intra-PL activity. Right: Representative histology showing the expression of somatic ChR2 in PL contralateral to the recording site (top) and SSFO expression in the MD (bottom). Scale bar in µm: 200. **b**, Example rasters and PSTHs of a putative excitatory PL neuron showing an evoked response to intra-PL activation alone (left) and no change with concurrent MD activation (right). Blue ticks mark the period of contralateral PL stimulation. **c**, Population quantification of effect in **b** (n = 151 excitatory PL neurons from 4 mice, ***p = 1 x 10^-15^, compared across groups, Wilcoxon signed-rank). **d**, Left: Same as in **a** except for stimulation of auditory thalamus (MGB) and measuring evoked responses in the auditory cortex (A1). Right: Representative histology of somatic ChR2 expression in A1 contralateral to the recording site (top) and SSFO expression in the MGB (bottom). Scale bar in µm: 200. **e**, An excitatory A1 neuron showing response to intra-A1 activation alone (left) and an amplification of its response with concurrent MGB activation (right). Blue ticks mark the period of contralateral A1 stimulation. **f**, Population quantification of effect in **e** (n = 196 neurons from 3 mice, p = 0.6802 (NS), compared across groups, Wilcoxon signed-rank test). **g**, ChR2 stimulation in PL and A1 respectively evoke comparable responses in the contralateral PL and A1 (n = 151 and 196 excitatory units recorded from the PL of 4 animals and the A1 of 3 animals respectively; p = 1x10^-5^, compared to baseline, p = 0.2532 (NS), across groups, Mann-Whitney U test). **h**, MD activation induced increase in baseline spike rates of PL inhibitory neurons is unaffected by concurrent suppression of PL VIP+ neurons (n = 48 neurons; ^+^p = 0.0243, ^++^p = 0.0086, compared to baseline, Mann-Whitney U test, p = 0.7555 (NS), compared across groups, Wilcoxon signed-rank test). **i**, Example of a putative PL excitatory neuron showing a response to intra-PL activation alone (top), which remains unaffected by concurrent suppression of PL VIP+ neurons (bottom). Blue tick marks the period of contralateral PL stimulation and yellow bar marks the duration of VIP+ inactivation. **j**, Quantification of effect in **i** (n = 151 neurons from 4 mice, ^+++^p = 1.0 x 10^-5^ (VIP int.),^+++^p = 1.0 x 10^-5^ (VIP sil.) compared to baseline; Mann-Whitney U test; p = 0.5956 (NS), compared across groups; Wilcoxon sign ranked test). **k**, Optical inactivation of PL VIP+ interneurons do not affect baseline spike rates of putative excitatory or inhibitory neurons in the PL (n = 385 excitatory (RS) and n = 98 inhibitory (FS) neurons from 4 mice; p=0.0955 (NS, RS); p=0.4933 (NS, FS), compared to baseline, Mann-Whitney U test). All statistical tests are two-tailed. For box plots **g**, **h**, **j**, **k** boundaries, 25–75th percentiles; midline, median; whiskers, minimum–maximum Source data.

Activating the mediodorsal thalamus (MD) in mice has two distinct effects on neural activity in the prefrontal cortex (PFC): amplification of local functional connectivity^7^ and suppression of spike rates^8^. To ask what the circuit mechanisms of these effects were, we first replicated them (Extended Data Fig. 1, Fig. 1), and confirmed that they were specific to this associative thalamocortical loop^8^ (Extended Data Fig. 1a–g). We noted that, in contrast to sensory systems^9^, the MD heavily targets cortical interneurons that are positive for vasoactive intestinal peptide (VIP^+^)^10^ and known to be important for input amplification through disinhibition^11^. Therefore, we asked whether MD-dependent amplification of PFC functional connectivity (Methods) was dependent on VIP^+^ interneurons. Indeed, suppressing VIP^+^ interneurons eliminated this MD effect (Fig. 1a–c, Extended Data Fig. 1i, j), but, notably, did not affect basal cortical spike rates (Fig. 1d, e, Extended Data Fig. 1h, k). The two MD effects were uncorrelated, suggesting mechanistic independence (Fig. 1f). As such, we hypothesized that the MD may contain two projections that differentially target prefrontal interneurons for independent control over input amplification and suppression (Fig. 1g). We also hypothesized that suppression may be carried out by parvalbumin positive (PV^+^) prefrontal interneurons, as several studies have shown robust activation of these interneurons by the MD^12^. The specific subdivision of the PFC that we focus on in this study is the prelimbic cortex (PL).

## Identifying genetic MD cell types

**Fig. 2:**
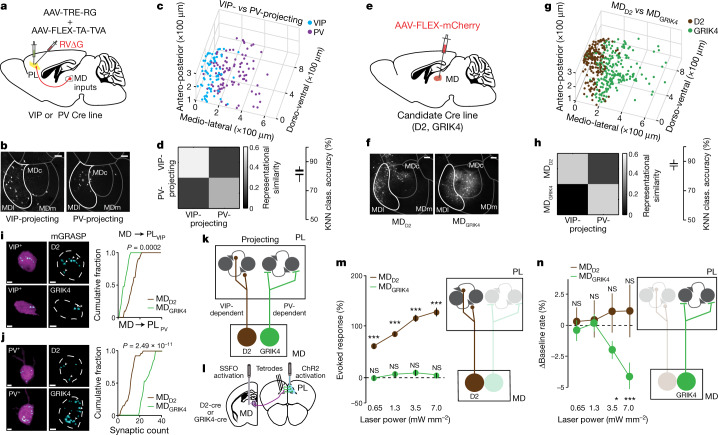
Two MD circuits for amplification and suppression of PFC activity. **a**, Prefrontal PV^+^ and VIP^+^ input mapping. **b**, MD neurons targeting VIP^+^ (left) and PV^+^ (right) interneurons occupy distinct MDl domains. Scale bars, 200 µm. **c**, Group summary for location of VIP- and PV-projecting MDl neurons (*n* = 73 VIP-projecting (7 mice) and *n* = 117 PV-projecting (4 mice)). **d**, KNN clustering and representational similarity analysis show robust separation. **e**, Labelling MD_D2_ and MD_GRIK4_ neurons using the corresponding Cre lines. **f**, MD_D2_ and MD_GRIK4_ neurons also occupy distinct anatomical locations. Scale bars, 200 ﻿μm. **g**, Group summary of MD_D2_ and MD_GRIK4_ neurons in MDl (*n* = 177 MD_D2_ neurons and 194 MD_GRIK4_ neurons from 3 mice each). **h**, MD_D2_ and MD_GRIK4_ locations show additional high representational similarity to VIP- and PV-projecting neurons, respectively. **i**, **j**, mGRASP labelling shows higher innervation of VIP^+^ neurons by MD_D2_ (**i**; *n* = 21 neurons from 3 D2-cre, *n* = 25 neurons from 3 GRIK4-cre mice, respectively; Kolmogorov–Smirnov) and higher innervation of PV^+^ neurons by MD_GRIK4_ (**j**; *n* = 27 neurons from 3 D2-cre, *n* = 32 neurons from 3 GRIK4-cre mice, respectively; Kolmogorov–Smirnov). Scale bars, 3 µm. **k**, Hypothesized circuit. **l**, Selective MD cell-type activation set-up. **m**, MD_D2_ but not MD_GRIK4_ amplify functional PL connectivity (*n* = 100 and *n* = 68 PL neural responses from 3 mice each for MD_D2_ and MD_GRIK4_, respectively; left to right: MD_D2_ *P* = 1.0 × 10^−5^ for all; MD_GRIK4_ *P* = 0.0599, 0.0789, 0.0575, 0.1311 (NS) for laser powers displayed; Mann-Whitney *U*, compared to baseline). **n**, MD_GRIK4_ but not MD_D2_ suppress PL neural spike rates (*n* = 1,257 and *n* = 697 putative excitatory PL neurons from 3 mice each; MD_D__2_ *P* = 0.184, 0.605, 0.579, 0.739 (NS); MD_GRIK4_ *P* = 0.298, *P* = 0.067, *P = 0.033, ***P = 1.61 × 10^−5^, respectively, for laser powers displayed; Mann-Whitney *U* compared to baseline). All statistical tests are two-tailed. Box plot parameters as in Fig. 1. Data are mean ± s.e.m. for **m**, **n**. Source data.

To investigate the anatomical circuitry for these two hypothesized MD projections, we performed monosynaptic rabies tracing from either VIP^+^ or PV^+^ interneurons in the PL. Notably, we found that MD neurons projecting to these two prefrontal interneuron types occupied distinct anatomical territories (Fig. 2a–d, Extended Data Fig. 3a–e). Given genetic variation across the mediolateral axis of the thalamus^13^, we reasoned that these thalamic projections may be genetically distinct.

**Extended Data Fig. 2:**
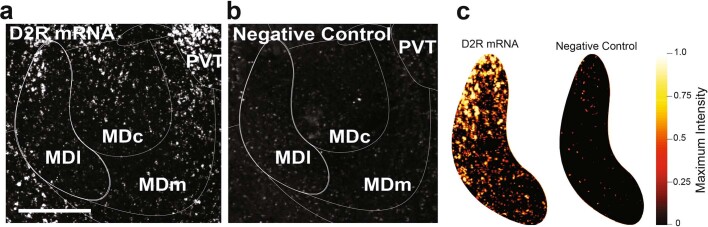
D2 receptor mRNA expression in the mouse MD. **a**, Representative histology showing the expression of D2 receptor mRNA in the lateral MD using fluorescent in situ hybridization. **b**, Negative control probe does not result in staining. Scale bar = 300 µm **c**, Heat map quantifying expression of D2 receptor mRNA across all MD sections (n = 8 sections for each condition from 2 mice).

A recent study in the paraventricular thalamus showed that the dopamine type 2 receptor (D2) distinguishes two subpopulations of functionally distinct thalamic projection neurons^14^. The MD is known to receive dopaminergic inputs^15^, and we found the mRNA expression of the D2 receptor to be reminiscent of the anatomical location of VIP-projecting MD neurons (Extended Data Fig. 2). Indeed, MD labelling in the D2-cre mice indicated that the D2^+^ genotype and the VIP-projecting one may be related (Fig. 2e, f).

**Extended Data Fig. 3:**
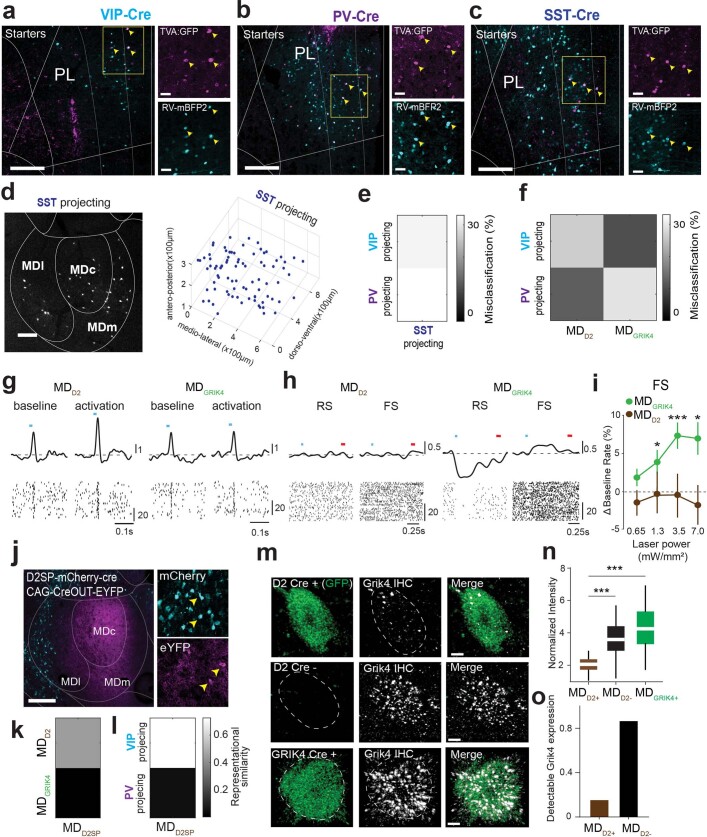
Supportive evidence for anatomical and functional segregation of the two MD cell types. **a-c**, Left: Starter neurons (arrowheads) in the PL of VIP-cre (**a**), PV-cre (**b**) and SST-cre (**c**) mice for monosynaptic retrograde tracing using rabies viruses. Right: Starter neurons identified by co-expression of TVA fused to GFP (top) and blue fluorescent protein from rabies viruses (bottom) in VIP, PV, and SST neurons, respectively. Scale bars in µm: 200 µm(left), 30 µm (right). **d**, Left: Representative images of MD neurons that monosynaptically target PL SST+ interneurons. Scale Bar in µm: 200. (Note a lack of preferential localization within the MDl) Right: 3D plot of the anatomical location of SST-projecting MDl neurons (n = 86 SST projecting neurons from 3 mice). **e**, Anatomical separation between SST-projecting MD neurons and VIP-/PV- projecting MD neurons quantified as high misclassification using KNN clustering. **f**, MD_D2_ and MD_GRIK4_ neuronal locations show low misclassification compared to VIP- and PV-projecting MD neurons respectively. **g**, Example of a PL neuron showing amplification of evoked responses through concurrent intra-PL and MD_D2_ optical stimulation (left), but not when intra-PL stimulation is combined with MD_GRIK4_ stimulation (right). **h**, Examples of excitatory (RS) and inhibitory (FS) PL neurons showing, respectively, suppression and increase in spike rates with optical activation of MD_GRIK4_ neurons but not with activation of MD_D2_ neurons. **i**, Parametric activation of MD_GRIK4_, but not MD_D2_, neurons increase spike rates of PL inhibitory neurons (n = 68 and n = 78 PL-FS neurons from 3 animals each of MD_D2_ and MD_GRIK4_ respectively; p = 0.874, For MD_GRIK4_ p = 0.556, *p = 0.0387, ***p = 9.36 x 10^-6^, *p = 0.0387 respectively for laser powers 0.65, 1.3, 3.5 and 7.0 mW/mm^2^; Mann-Whitney U test compared to baseline). **j**, left: D2 specific promoter (D2SP) driven expression of mCherry + (CreON) and co-expression of EYFP (CreOFF) in Cre-negative neurons using a Cre - Out intersectional strategy labels two populations similar to D2-cre and Grik4-cre, but in WT animal. Right: Magnified images showing mCherry (D2SP+) and eYFP (Cre negative) neurons. Scale bar = 200 µm **k**, Consistent anatomical similarity between MD_D2SP_ and MD_D2_ populations and a corresponding segregation between MD_D2SP_ and MD_GRIK4_ neurons, quantified using representational similarity analysis (n = 95 cells from 2 animals for MD_D2SP_). **l**, A comparable similarity and segregation as shown in (**k**) is found when comparing MD_D2SP_ neurons to VIP-projecting and PV projecting neurons. **m**, top row: MD_D2_ Cre-expressing neurons (MD_D2+_) labelled with GFP have extremely sparse Grik4 protein expression (IHC) compared to MD_D2_ Cre-negative (MD_D2_- neurons (middle row) or MD_GRIK4_ expressing neurons (bottom row). Scale bar = 3 µm **n**, Quantification of data (n = 116 MD_D2+_, 106 MD_D2-_ and 124 MD_GRIK4_ neurons from 2 animals, demonstrating substantial Grik4 immunolabelling overlap between D2- and Grik4+ neural populations (not significantly different), but both being different from the D2+ population. ***p = 0.0001 for both comparisons, Kruskal Wallis test). **o**, Direct comparison of Grik4 immunolabelling across D2+ and D2- neurons (thresholded by the lowest 10th percentile of this analysis puts an upper bound estimate of 15% overlap between the D2+ and Grik4+ population. ‘positive control’ Grik4+ neurons). All statistical tests are two-tailed. For box plot **n** boundaries, 25–75th percentiles; midline, median; whiskers, minimum–maximum. Data are presented as mean ± SEM for **i** Source data.

To identify a potential genotype for the PV-projecting MD neurons, we took note of a previous study that used the kainate receptor, GRIK4, to label a population of MD neurons that drove feedforward inhibition^16^, mediated through PV^+^ interneurons^17^. MD labelling in GRIK4-cre mice (Fig. 2e) resulted in a pattern resembling the PV projection identified earlier (Fig. 2f). In addition, the D2^+^ (MD_D2_) and GRIK4^+^ (MD_GRIK4_) neurons could reliably be anatomically separated across mice, in a manner similar to VIP- and PV-projecting neurons (Fig. 2g, h). We confirmed the correspondence between this anatomical connectivity phenotype and its genetic identity through cross-validation (Fig. 2h, Extended Data Fig. 3f).

**Extended Data Fig. 4:**
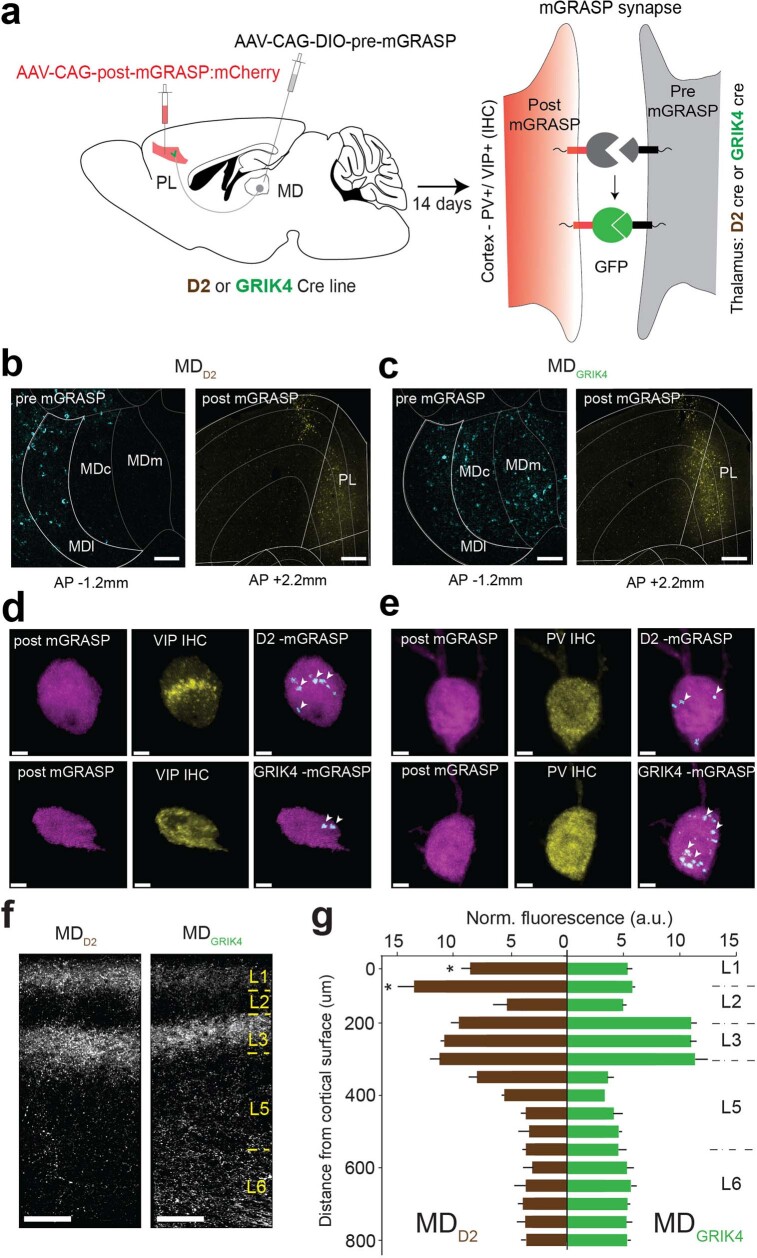
mGRASP and synaptophysin labelling provide evidence for output segregation of the two MD cell types. **a**, Cartoon depicting strategy to label cell type specific MD→PL thalamocortical synapses using mGRASP. The pre mGRASP component is virally expressed in MD_D2_ or MD_GRIK4_ neurons in the respective Cre lines while the post mGRASP component is ubiquitously expressed in the PL. MD_D2_ or MD_GRIK4_ specific mGRASP synapses onto VIP vs PV neurons in the PL are identified by immunohistochemistry guided detection of PV and VIP neurons expressing post mGRASP in the PL. **b–c**, Left: Representative images of MD_D2_ (**b**) and MD_GRIK4_ (**c**) neurons expressing pre mGRASP in the MD of D2-cre and GRIK4-cre mice respectively. Right: Ubiquitous expression of post mGRASP+ neurons detected by TdTomato fluorescence in the PL of D2-cre (**b**) and GRIK4-cre (**c**) mice. Scale bar in µm: 200. **d**, Left to right: Examples of PL VIP+ neurons showing post mGRASP expression (magenta), VIP expression detected via immunohistochemistry (yellow) and mGRASP+ synapses from MD_D2_ (cyan dots, top row) or MD_GRIK4_ (cyan, dots, bottom row) neurons. **e**, Same as in **d**, for PL PV+ neurons. Scale bars in µm: 3 µm. **f**, Representative images showing layer-wise termination of synapses from MD_D2_ (left) and MD_GRIK4_ (right) neurons in the PL, labelled with virally expressed GFP fused to synaptic protein (synaptophysin). Scale bar in µm: 100. **g**, MD_D2_ neurons terminate in L1 of the PL with a higher frequency compared to MD_GRIK4_ neurons (n = 12 sections each from 3 D2-cre and 3 GRIK4-cre mice, *p = 0.0253, *p = 0.039, two-tailed Mann-Whitney U test comparing 50 µm bins from the pial surface across groups). All statistical tests are two-tailed. Data are presented as mean ± SEM for **g** Source data.

To further test the hypothesis that the two thalamic projections map onto distinct genetic identities, we used a synaptic labelling technique: mammalian GFP reconstitution across synaptic partners (mGRASP)^18^ (Extended Data Fig. 4). After Cre-dependent presynaptic mGRASP injection into the MD of either D2-cre or GRIK4-cre mice, and pan-neuronal postsynaptic mGRASP in the PL, we quantified the pattern of synaptic innervation of PV^+^ and VIP^+^ neurons (identified by immunohistochemistry) across these preparations (Extended Data Fig. 4a–c). We found that the MD_D2_ population preferentially targeted VIP^+^ neurons (Fig. 2i, Extended Data Fig. 4d), whereas the MD_GRIK4_ preferentially targeted PV^+^ neurons (Fig. 2j, Extended Data Fig. 4e). This finding was independently supported by synaptophysin-based labelling; MD_D2_ neurons preferentially targeted layer I (Extended Data Fig. 4f, g), where VIP^+^ neurons are known to be enriched^10^. Collectively, these experiments indicated that PL amplification and suppression may indeed be under the control of genetically distinct MD thalamic cell types (Fig. 2k).

To directly test this idea, we selectively activated either MD_D2_ or MD_GRIK4_ neurons (Fig. 2l), and found that the former—but not the latter—resulted in amplification of functional PL connectivity (Fig. 2m, Extended Data Fig. 3g), whereas the opposite dependence was true for spike rate suppression (Fig. 2n, Extended Data Fig. 3h, i). These experiments definitively show that the MD contains two genetically distinct projections that independently control PL activation and suppression. Of note, MD_D2_ and MD_GRIK4_ segregation was independently verified using a viral strategy (Extended Data Fig. 3j–l), and GRIK4 immunohistochemistry allowed us to estimate their overlap to be 5–15% (Extended Data Fig. 3m–o).

**Extended Data Fig. 5:**
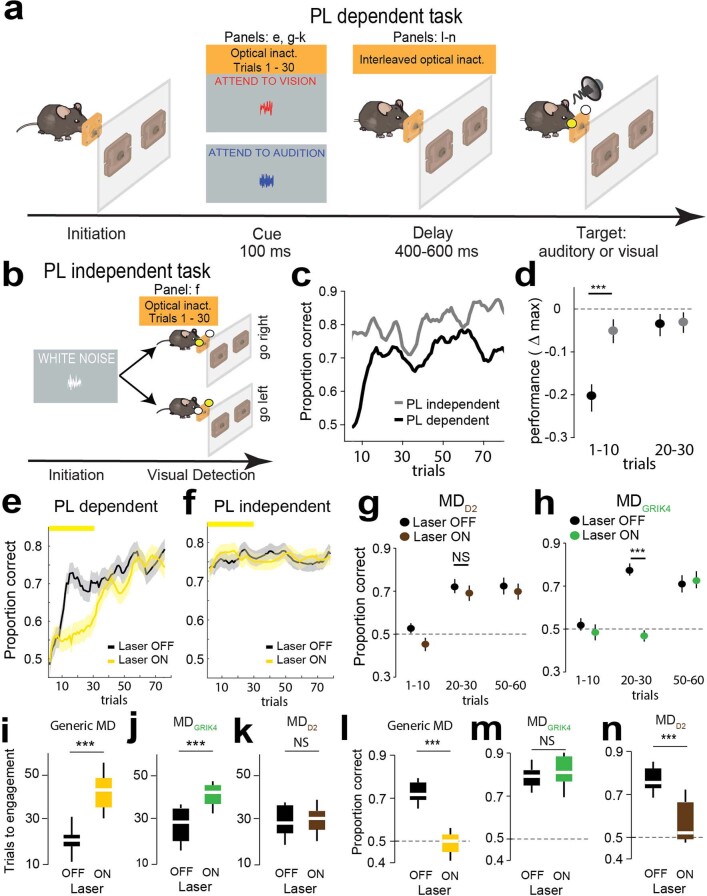
Differential engagement of the two MD cell types in a PL-dependent behaviour. **a**, Schematic illustration of PL dependent attention control task (see Methods). In brief, on each trial, animals have to remember a 100ms HP or a LP auditory cue over a delay period to execute the corresponding rule (HP - attend to audition vs LP – attend to vision) and make a choice to either follow a target stimulus (auditory vs visual) to collect a milk reward. Orange bars highlight the two epochs where MD is optically inhibited across the experiment types **b**, Illustration of PL independent task with a 2AFC design, where animals have to respond to the side a LED light target was presented (without a distractor). **c**, Example sessions plotting the performance of an animal in the PL dependent (black) and PL independent (grey) versions of the task. **d**, Well trained animals show a daily ramp-up of performance in the first 30 trials of the PL dependent (but not PL independent) task, starting from chance (trials 1 to 10) and progressing onto performance > 0.7 proportion correct (‘task engagement’, trials 20 to 30) (n = 12 sessions over 4 mice, ***p = 0.0002,). **e**, **f**, optical MD inactivation (yellow) during the cueing period of the first 30 trials in a session prolongs the ramp up to task engagement in the PL dependent task (**e**) but not the PL independent task (n = 12 sessions over 4 mice, p = 0.0008, Kolmogorov-Smirnov test). **g**, Optical inactivation of MD_D2_ neurons have no effect on number of trials taken to task engagement in PL dependent attention control task (n = 12 sessions over 4 D2-cre mice, p = 0.466 (NS), comparing across laser ON vs laser OFF sessions). **h**, Optical inactivation of MD_GRIK4_ neurons increase the number of trials required to reach task engagement in the attention control task (n = 12 sessions over 4 GRIK4-cre mice, ***p = 2.2 x 10^-6^, comparing across laser ON vs laser OFF sessions). **i**, Optical MD inactivation during the cueing period of the first 30 trials delays task engagement (performance at > 0.7 proportion correct; n = 12 sessions over 4 animals, ***p= 1.5 x 10^-6^;). **j-k**, Optical MD_GRIK4_ (but not MD_D2_) inactivation recapitulates the effect in b (n = 12 sessions each from 4 D2-cre and 4 GRIK4-cre mice; ***p = 2.9 x 10^-5^, p = 0.7657 (NS);). **l**, Optical MD inactivation in the delay period of <20% of trials diminish performance on those trials (n = 12 sessions over 4 mice, ***p=7.4x10^-7^;). **m-n**, Optical inactivation of MD_D2_ (but not MD_GRIK4_) recapitulates the effect in **e**. (n = 12 sessions each from 4 D2-cre and 4 GRIK4-cre mice respectively; p = 0.3394 (NS), ***p = 2.95 X 10^-6^). two-tailed Mann-Whitney U test. All statistical tests are two-tailed. For box plots **i-n** boundaries, 25–75th percentiles; midline, median; whiskers, minimum–maximum. Data are presented as mean ± SEM for **d–h** Source data.

To test whether these two cell types differentially engage in MD–PL-dependent behaviour, we leveraged an attentional control task that can distinguish MD enhancement of PL activity to maintain attentional control signals^7,19^, and MD suppression of PL activity to enable task switching^12,20^, or engagement (Extended Data Fig. 5a–f). Selective MD_D2_ inactivation diminished the former, whereas selective MD_GRIK4_ inactivation diminished the latter (Extended Data Fig. 5g–n, Supplementary Note 1).

## Mouse MD tracks task uncertainty

**Fig. 3:**
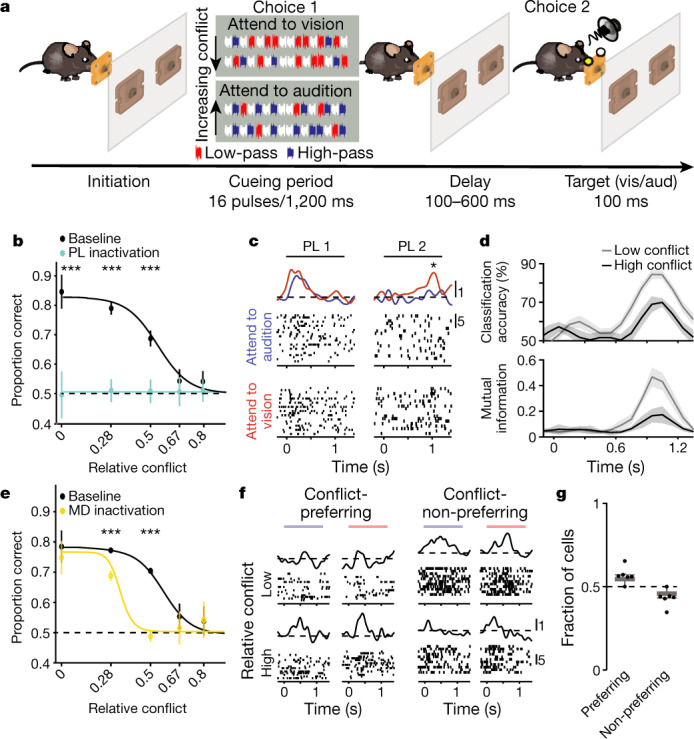
Task input uncertainty engages the mouse MD. **a**, Task schematic (see text). **b**, PL inactivation (blue) diminishes performance regardless of uncertainty level (*n* = 17 sessions, 5 mice; ***P < 7.72 × 10^−4^; chi-squared). **c**, Putative excitatory PL neurons showing responses during the cueing period, with the later neuron showing selectivity to the attentional choice (*P = 0.0157; Mann-Whitney *U*). **d**, Population decoding (top) and mutual information (bottom) show choice selectivity (*n* = 1,112 neurons from 7 mice). Both measures are modulated by uncertainty. **e**, MD inactivation (yellow) diminishes performance as a function of uncertainty (*n* = 56 sessions, 6 mice; ***P < 1.43 × 10^−13^; chi-squared). **f**, Example task-relevant MD neurons, one conflict-preferring and one conflict-non-preferring, both exhibiting little choice selectivity. **g**, Relative fraction of the MD neural functional types (*n* = 2,669 neurons from 7 mice). All statistical tests are two-tailed. Box plot parameters as in Fig. 1. Data are mean ± s.e.m. for **b**, **e** and mean ± 95% confidence interval (CI) for **d**. Source data.

**Extended Data Fig. 6:**
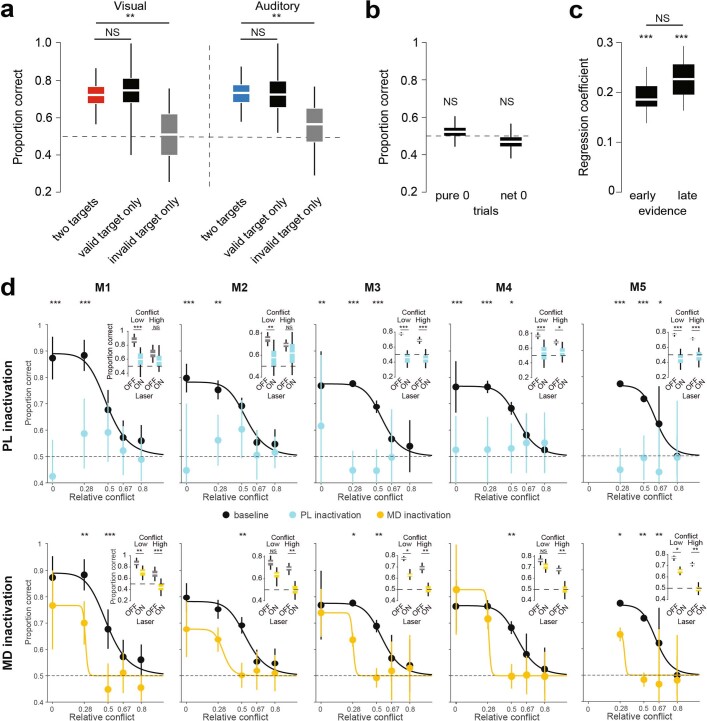
Controls that clarify behavioural strategy and weighing of evidence in the attentional control task with input uncertainty. **a**, Behavioural validation of animals using correct task execution strategies (see Fig. 3a). Omitting the distractor on a subset of interleaved trials (15%, valid target only) during choice 2 (Fig. 3a) had no effect on behaviour (n = 41 sessions over 6 mice; p = 0.859 (NS, visual); p = 0.728 (NS, auditory); Mann-Whitney U test). Omitting the target on a similar subset of trials (invalid target only) reduced performance accuracy down to chance level (**p = 0.00649 (visual); **p = 0.00216 (auditory); Mann-Whitney U test). Combined, these data indicate that animals did not adopt a pro-anti strategy based on a single target (vision or audition). **b**, Average performance on uninformative trials is comparable when the underlying sequences are composed of only broadband white noise pulses (n = 11 sessions over 6 mice; pure 0, p = 0.353 (NS), binomial test) or informative cues with zero overall net evidence (net 0, p = 0.690 (NS), binomial test). **c**, Regression analysis shows that evidence in the early half and the late half of the cueing sequence contribute equally to animal choice behaviour (n = 54 sessions over 6 mice; early ***p = 2.98 x 10^-7^, t = 5.12 compared to 0; late ***p = 5.03 x 10^-10^, t = 6.22 compared to 0; early vs late p = 0.368 (NS), t = 0.901; degree of freedom=3946; student’s t-test). **d**, Full psychometric functions of individual mice in the conflict-driven input uncertainty task. Performance accuracy in the distributed cue task with input uncertainty due to cueing conflict, separated by animals. For each animal, performance accuracy consistently diminishes with increased cueing conflict (black traces, top and bottom row), while optical PL inactivation (blue traces, top row) during the cueing period strongly suppresses performance regardless of input uncertainty (M1: ***p = 1.60 x 10^-10^ (relative conflict = 0), ***p = 1.89 x 10^-6^ (relative conflict = 0.28); M2: ***p = 6.40 x 10^-8^ (relative conflict = 0), **p = 0.00366 (relative conflict = 0.28); M3: **p = 0.00149 (relative conflict = 0), ***p = 2.38x10^-6^ (relative conflict = 0.28), ***p = 2.85 x 10^-4^ (relative conflict = 0.5); M4: ***p = 1.19 x 10^-4^ (relative conflict = 0), ***p = 4.83 x 10^-4^ (relative conflict = 0.28), *p = 0.0361 (relative conflict = 0.5); M5: ***p = 4.18 x 10^-6^ (relative conflict = 0.28), ***p = 8.86 x 10^-4^ (relative conflict = 0.5), *p = 0.0130 (relative conflict = 0.67); chi-squared test). In contrast, Optical MD inactivation (yellow traces, bottom row) during the cueing period reduces performance more strongly on high conflict trials than on low conflict trials, consistently across animals (M1: **p = 0.00345 (relative conflict = 0.28), ***p = 2.27x10^-4^ (relative conflict = 0.5); M2: p = 0.111 (NS; relative conflict = 0.28), **p = 0.00676 (relative conflict = 0.5); M3: *p = 0.0208 (relative conflict = 0.28), **p = 0.00556 (relative conflict = 0.5); M4: p = 0.426 (NS; relative conflict = 0.28), **p = 0.00651 (relative conflict = 0.5); M5: *p = 0.0486 (relative conflict = 0.28), **p = 0.0.00107 (relative conflict = 0.5), **p = 0.00227 (relative conflict = 0.67); chi-squared test). Inset in each panel highlights the effect of PL/MD inactivation on trials with low (0.28) and high (0.5) conflict. All statistical tests are two-tailed. For box plots **a-c** and insets in **d**, boundaries, 25–75th percentiles; midline, median; whiskers, minimum–maximum. Data are presented as mean ± SEM for **d** Source data.

We next turned our attention to asking whether these cell types contribute to a domain that may generalize to human cognition. Studies of the human brain have indicated a particular role for the MD in decision-making that scales with the degree of task input uncertainty^1,21^. Therefore, we reasoned that incorporating input uncertainty into a task requiring MD–PL interaction in mice could achieve this goal. Consequently, we modified an attentional control task^7^ by parametrizing its cueing component (Fig. 3a, Methods). Specifically, on each trial a mouse was presented with a sequence of sixteen sound pulses (different mixtures of high-pass (HP, ‘attend to audition’), low-pass (LP, ‘attend to vision’) or broadband white noise (‘blank’)). Target selection was tied to the rule with the highest number of corresponding pulses on each sequence, and the ambiguity was mainly controlled by the conflict between HP and LP pulses. Multiple controls were incorporated to ensure that mice were adopting an attentional selection strategy (Extended Data Fig. 6a) and that they interpreted broadband white noise pulses as ‘blanks’ (Extended Data Fig. 6b). Finally, regression analysis further validated that the mice were weighing evidence in the early and late halves of the cueing period equivalently (Extended Data Fig. 6c).

**Extended Data Fig. 7:**
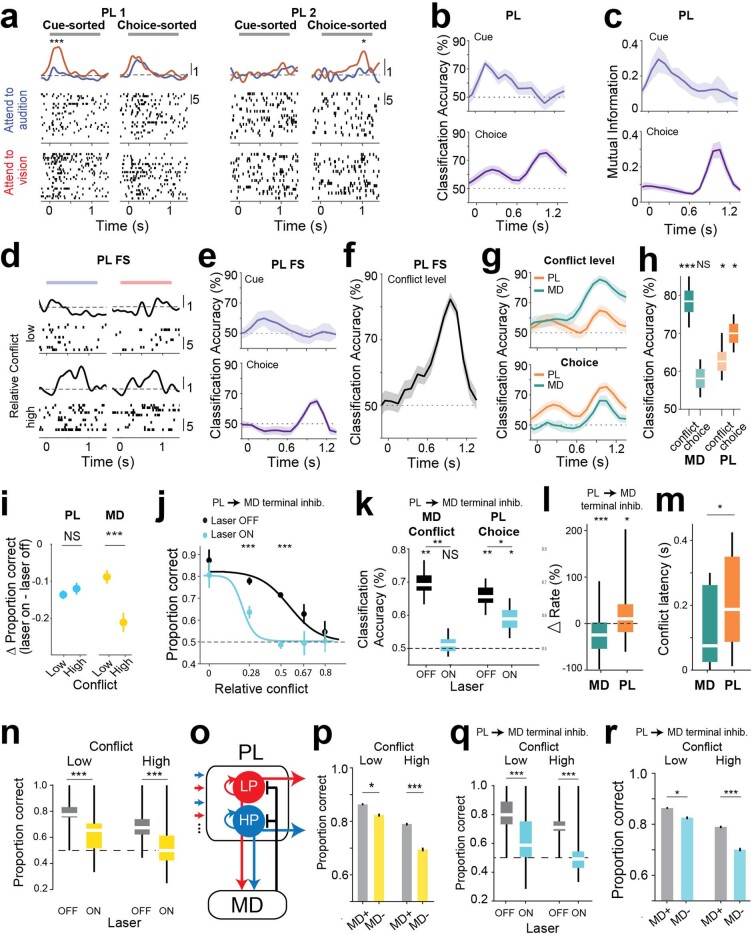
Extended analysis and relevant controls of PL RS and FS cells, and differential encoding of task relevant variables across the MD and PL. **a**, Two example excitatory PL neurons shown in Fig. 3c, sorted by momentary cue (cue-sorted) and attentional choice (choice-sorted). The earlier-responding neuron (left) shows selectivity to momentary cue and the later-responding neuron (right) shows selectivity to the attentional choice (***p = 6.17x10^-4^; *p = 0.0157; Mann-Whitney U test). In contrast, there are weak choice selectivity for the earlier-responding neuron and weak cue selectivity for the later-responding neuron. **b**, Quantification of PL population selectivity to momentary cue (top) and attentional choice (bottom) using linear decoding (n=1112 neurons from 7 mice). Note that population cue selectivity is strong early on but gradually decreases, while population choice selectivity peaks late in the cueing period. **c**, Quantification of PL population selectivity to momentary cue (top) and attentional choice (bottom) using mutual information. **d**, Example putative inhibitory fast spiking neuron, showing higher firing rate for trials with high conflict, and little attentional choice selectivity. This neuron shows similar selectivity to the example conflict-preferring MD neuron (Fig. 3f). **e**, Quantification of selectivity of putative inhibitory fast spiking neuron population in PL to momentary cue (top) and attentional choice (bottom) using linear decoding (n = 104 neurons from 7 mice). The selectivity for both cue and choice are weak compared to the putative excitatory neuron population (**b**). **f**, Quantification of conflict selectivity of putative inhibitory fast spiking neuron population in PL using linear decoding, showing strong conflict selectivity. **g**, Quantification of PL and MD population selectivity to conflict (top) and attentional choice (bottom) using linear decoding. MD population demonstrates strong conflict and weak choice selectivity (n = 2669 neurons from 7 mice), while PL population demonstrates strong choice and weak conflict selectivity. **h**, Choice modulated PL neurons demonstrate moderate conflict selectivity (*p = 0.046 choice, *p = 0.02 choice; permutation test). In contrast, conflict modulated MD neurons have no choice selectivity (***p = 0.0005 conflict, p=0.695 choice (NS); permutation test). n=50 most modulated neurons each. **i**, Optical inactivation of PL and MD result in distinct impairments in task performance across different levels of conflict driven input uncertainty. The magnitude of optical PL inactivation is titrated to match the task performance on low conflict trials with optical MD inactivation. PL inactivation results in comparable impairments in performance accuracy across low and high conflict trials, while MD inactivation has a stronger effect on high conflict trials compared to low conflict trials (n =13 sessions from 5 mice; ***p<0.001, Mann-Whitney U test). **j**, MD deafferentiation during the cueing period impairs performance more strongly on high conflict trials than on low conflict trials (n = 37 sessions over 6 mice; ***p=4.84 x 10^-6^ (relative conflict = 0.28); ***p= 1 x 10^-15^ (relative conflict=0.5); chi-squared test), similar to optical MD inactivation (Fig. 3e). **k**, Quantification of MD population conflict selectivity and PL population choice selectivity. MD deafferentiation annihilates MD conflict classification accuracy and weakens PL choice classification accuracy (n = 386 putative excitatory neurons and n = 666 MD neurons from 3 mice; **p = 0.005 (MD conflict, Laser OFF); p = 0.96 (NS, MD conflict, Laser ON); **p = 0.0042 (MD conflict, Laser OFF vs ON); **p = 0.005 (PL choice, Laser OFF); *p = 0.048 (PL choice, Laser ON); *p = 0.012 (PL choice, Laser OFF vs ON); permutation test). **l**, MD deafferentiation result in lowered firing rate in MD (***p = 9.32x10^-11^) and higher firing rate in PL excitatory neurons (*p = 0.0231; Wilcoxon signed-rank test). Data is pooled over conflict-preferring MD neurons (n = 201 neurons), and choice-selective PL neurons (n = 85 neurons,). **m**, MD neurons respond to conflict earlier in time compared to PL excitatory neurons (*p = 0.0289; Mann-Whitney U test). Shown are the latency to reach maximum regression coefficient after the conflict signal emerges. **n**, Data in Fig. 3e reorganized, highlighting the effect of MD inhibition on trials with low (0.28) and high (0.5) conflict. **o**, **p**, A mean-field neural model, which describes choice accumulation in the PL recaptures experimental data in **n** (n = 2,000 trials, *p = 0.0137; ***p = 1.18 x 10^-6^; chi-squared test). **q**, Data in **j** reorganized, highlighting the effect of optical inhibition of PL→MD terminals on trials with low and high conflict. **r**, Mean-field neural model (see Extended Data Fig. 7o) captures the effect of inhibition of PL→MD terminals on task performance (n = 2,000 trials, *p = 0.0189; ***p = 4.90 x 10^-6^; chi-squared test). All statistical tests are two-tailed. For box plots **h**, **k-n**, **q** boundaries, 25–75th percentiles; midline, median; whiskers, minimum–maximum. Data are presented as mean ± SEM for **i**, **j**, **p**, **r**, and mean ± CI for **b**, **c**, **e-g** Source data.

Inactivation of the PL during the cueing period diminished performance regardless of cueing uncertainty (Fig. 3b, Extended Data Fig. 6d). Electrophysiological recordings provided a putative explanation; PL neurons showed activity patterns consistent with transforming the task inputs to an attentional choice (Fig. 3c, d, Extended Data Fig. 7a–c). Notably, the rates of rise of attentional choice signals were modulated by uncertainty (Fig. 3d), indicating that PL ensembles may be integrating incoming cues into an attentional choice at a rate commensurate with input reliability. Consistent with this notion, putative inhibitory prefrontal fast spiking neurons showed modulation of spike rate by input uncertainty (Extended Data Fig. 7d–f). This finding gives rise to the notion that input uncertainty (which here we control through cueing conflict), engages prefrontal inhibition to modulate the speed of the cue-to-choice transformation.

Given the role of the MD in driving prefrontal inhibition^12,20^, and the human findings about its activity scaling with task input uncertainty^1^, we asked whether the MD was causally involved in the task. In contrast to the PL, MD inactivation during the cueing period did not cause a uniform detrimental effect in behavioural performance. Specifically, its effect scaled with the level of input uncertainty (Fig. 3e, Extended Data Fig. 6d). The effect of optical MD inactivation was not simply a weaker form of PL inactivation (Extended Data Fig. 7i). Multi-electrode recordings provided insight into its causal engagement; MD neurons showed a high degree of specialization for input uncertainty, with some neurons showing a preference to trials with high conflict, and others to low conflict (Fig. 3f, g, Extended Data Fig. 7g). Critically, although relative conflict could be decoded from the PL, that signal was carried by the same neurons that encoded the attentional choice, standing in sharp contrast to the specialization seen in the MD (Extended Data Fig. 7g, h).

We asked whether this specialized encoding of input uncertainty could be causal to scaling prefrontal inhibition (Extended Data Fig. 7d). Because associative thalamic areas like the MD may integrate their cortical inputs to generate such ‘summary statistic’ type signals^22,23^, we first tested whether optical deafferentiation of the MD by inhibiting PL terminals would diminish the encoding of conflict or uncertainty signals. Indeed, MD deafferentiation diminished conflict MD encoding (Extended Data Fig. 7k). Although this manipulation diminished behavioural performance (Extended Data Fig. 7j, q) and choice encoding in the PL (Extended Data Fig. 7k), it resulted in an overall increase in spike rates (Extended Data Fig. 7l), consistent with the MD primarily influencing PL cue-to-choice transformation through cortical inhibition in the current version of the task (Extended Data Fig. 7m).

**Extended Data Fig. 8:**
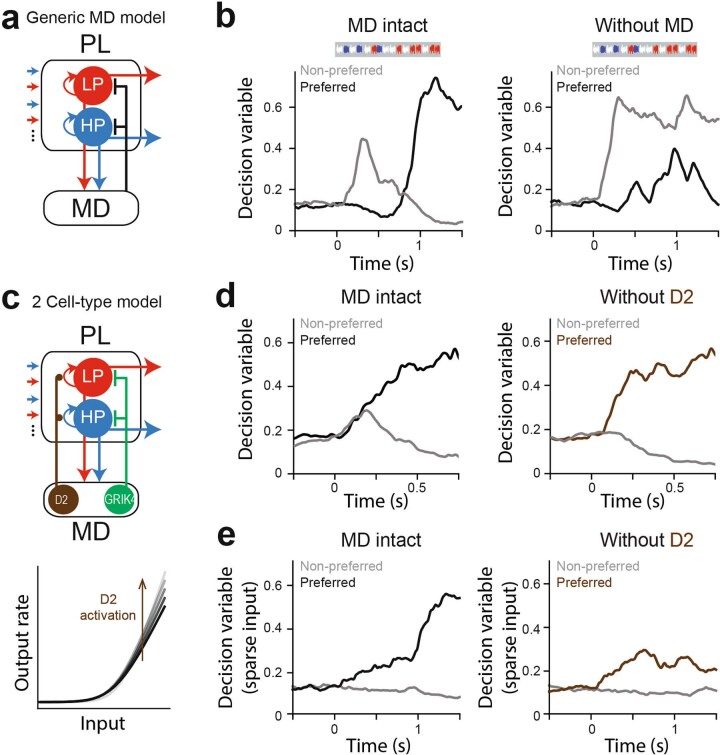
Basic and extended mean-field models. **a**, Schematic of the mean-field neural model that describes generic MD inactivation results (see Extended Data Fig. 7o). The model describes two PL populations that receive separate inputs corresponding to the cues in favour of the two attentional rules (HP - attend to vision or LP – attend to audition). Each population has strong recurrent self-excitation and net inhibition on the other population. The MD component of the model receives inputs from the PL (see Extended Data Fig. 7) and is activated by conflict to inhibit the two PL populations. **b**, Example model decision variables in a trial early biased to the wrong attentional choice, demonstrating how MD-mediated suppression may improve performance of the model. When MD is intact (left), strong early evidence to the wrong choice (high-pass in this example; cueing sequence in inset) increases the decision variable of the non-preferred population early on, but the preferred population prevails when the preferred stimulus dominates in the latter half of the cueing sequence. On the other hand, in the absence of MD conflict-driven suppression of cue integration in the PL (right), the early non-preferred inputs drive the non-preferred population to maintain high activity, suppressing the preferred population’s response to late inputs. **c**, Schematic of the mean-field neural model incorporating the two cell types, where MD_GRIK4_ is conflict-activated and suppresses PL, and MD_D2_ is conflict-suppressed and amplifies PL recurrence. MD_D2_ results in enhanced gain of the PL input-output function (bottom). **d**, Example model decision variables for high conflict trials, with (left) and without (right) MD_D2_. Increased PL recurrence due to MD_D2_ results in larger response to input cues. However, the effect is less pronounced for preferred cues as the population activity and decision variable saturate with inputs. As a result, the larger response to input cues asymmetrically favours the non-preferred population, and the separation between preferred and non-preferred activity is larger without MD_D2_ (shown are median over 1,000 trials). **e**, Example model decision variables for low signal sparse trials (Fig. 4), with (left) and without (right) MD_D2_ module. Increased PL recurrence due to MD_D2_ allows amplified response of the preferred population to sparse input cues, but minimally affects the non-preferred population which receives no input cues. As such, MD_D2_ results in a larger separation between preferred and non-preferred activity (shown are median over 1,000 trials).

To gain formal computational insight into this process, we built a neural model to study MD–PL interaction when inputs are conflicting (Extended Data Fig. 7o, Methods). This model was able to reproduce experimental data (Extended Data Fig. 7n, p–r), and provided insight into how the choice signal may be accumulated over time, and how MD-mediated suppression may slow it down when task inputs are conflicting and thereby unreliable (Extended Data Fig. 8a, b).

## MD types engage differently if inputs conflict

**Fig. 4:**
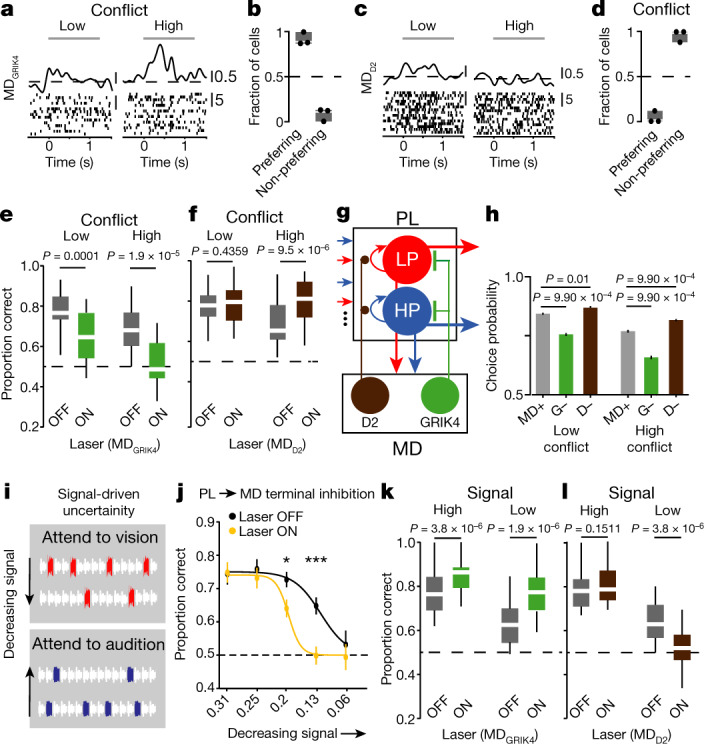
The two thalamic cell types are engaged by different task input statistics. **a**, Example tagged MD_GRIK4_ neuron recorded in the task. **b**, Tagged MD_GRIK4_ neurons are more likely to be conflict-preferring (*n* = 17 neurons from 3 mice; *P* = 0.0042; binomial). **c**, Example tagged MD_D2_ neuron recorded in the task. **d**, Tagged MD_D2_ neurons are more likely to be conflict-non-preferring (*n* = 20 neurons from 3 mice; *P* = 4.0 × 10^−5^; binomial). **e**, MD_GRIK4_ suppression recapitulates generic MD suppression (*n* = 20 sessions from 4 GRIK4-cre mice; Wilcoxon signed-rank). **f**, MD_D2_ inactivation enhances performance accuracy on trials with high cueing conflict (*n* = 20 sessions from 4 D2-cre mice; Wilcoxon signed-rank). **g**, Expanded neural model with two MD cell types. **h**, The two-cell-type model captures experimental data (*n* = 2,000 trials, chi-squared). MD+, MD intact; G−, without GRIK4; D−, without D2. **i**, Stimulus configuration for sparseness-driven uncertainty. **j**, Performance accuracy is modulated by cueing sparseness, and optical MD deafferentiation diminishes performance on trials with higher cueing sparseness (*n* = 25 sessions, 4 mice; *P = 0.0222, ***P = 1.02 × 10^−4^; chi-squared). **k**, MD_GRIK4_ inactivation improves performance accuracy on both high and low signal trials (*n* = 20 sessions from 4 GRIK4-cre mice; Wilcoxon signed-rank). **l**, Optical MD_D2_ inactivation recapitulates optical generic MD deafferentiation (*n* = 20 sessions from 4 D2-cre mice; Wilcoxon signed-rank). All statistical tests are two-tailed. Box plot parameters as in Fig. 1. Data are mean ± s.e.m. for **h**, **j**. Source data.

**Extended Data Fig. 9:**
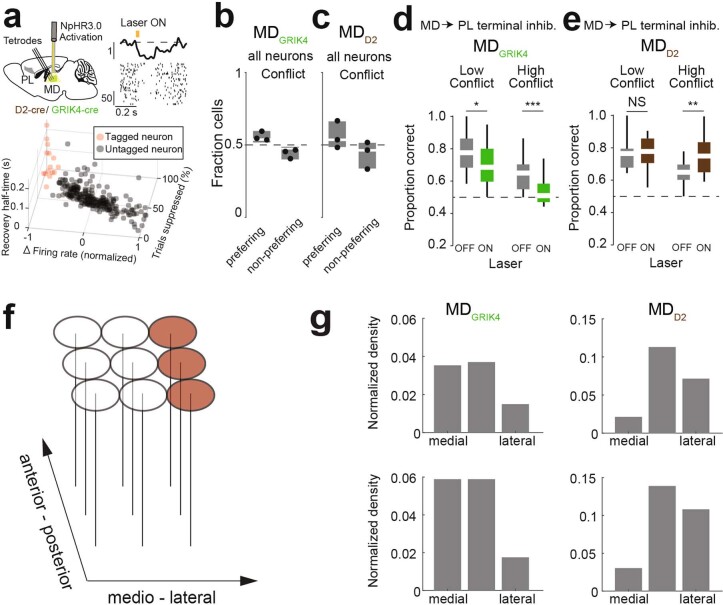
Untagged neurons in the tagging experiments are no different than generic recordings, and optical inhibition of terminals of the two cell types replicates cell body inactivation. **a**, (Top Left) Schematic of optogenetic tagging and identification of MD_D2_ and MD_GRIK4_ neurons. MD_D2_ or MD_GRIK4_ neurons are tagged with NpHR3.0 and identified via light activated spike rate suppression. (Bottom) Example tagged neuronal response to NpHR3.0 activation. (Right) Tagged neurons from one mouse (red) are identified using k-means clustering (features: change in firing rate, proportion of trials suppressed, and half-time to recover from suppression (n = 262 total number of neurons). **b**, Relative fraction of all MD neurons from GRIK4-cre mice that are conflict-preferring vs. non-preferring are comparable to that of wild-type animals (Fig. 3g) (n = 91 neurons from 3 mice; p = 0.429 (NS), chi-squared test). Note that tagged MD_GRIK4_ neurons are significantly more conflict-preferring compared to the whole population (Fig. 4b) (p = 0.0175; chi-squared test). **c**, Relative fraction of all MD neurons from D2-cre mice that are conflict-preferring vs. non-preferring, are also comparable to that of wild-type animals (Fig. 3g) (n = 95 neurons from 3 mice; p = 0.166 (NS), chi-squared test). Note, that tagged MD_D2_ neurons are significantly more conflict-non-preferring (Fig. 4d) (p=1.34 x 10^-4^; chi-squared test). **d**, Optical inhibition of MD_GRIK4_ terminals in the PL recapitulates the loss in task accuracy across low and high conflict trials as seen with optical MD_GRIK4_ inactivation (Fig. 4e; n = 20 sessions over 4 GRIK4-cre mice, *p = 0.0199, ***p = 0.0002; Mann-Whitney U test). **e**, Optical inhibition of MD_D2_ terminals in the PL enhances performance accuracy on trials with high cueing conflict, similar to the effect of optical MD_D2_ inactivation (Fig. 4f; n = 20 sessions over 4 D2-cre mice, p = 0.3941 (NS), **p = 0.0023; Mann-Whitney U test. **f**, Schematic of micro-drive bottom piece and the 3x3 grid organization of the tetrode array for MD recordings. **g**, Summary of the density of tagged neurons on the medial-lateral axis separated by animal. We show the result for 2 Grik4-cre (top and bottom) and 2 D2-cre animals ((top and bottom rows) that have enough numbers of tagged neurons. All statistical tests are two-tailed. For box plots **b – e** boundaries, 25–75th percentiles; midline, median; whiskers, minimum–maximum Source data.

Our results showed that MD_GRIK4_ neurons preferentially innervate PL PV^+^ neurons and that their activation inhibits baseline PL activity (Fig. 2n). Also, our neural model suggested that conflict-tracking in the MD drives PL inhibition to slow down cue integration when the inputs are less reliable (uncertain; Extended Data Fig. 7p). Thus, we hypothesized that conflict-tracking (or preferring) neurons may be GRIK4^+^. Indeed, optical tagging of MD_GRIK4_ neurons (Extended Data Fig. 9a) revealed that they were primarily conflict-preferring (Fig. 4a, b). By contrast, optically tagged MD_D2_ neurons showed the opposite functionality (Fig. 4c, d). Notably, non-tagged neurons in both of these preparations showed selectivity patterns consistent with generic MD recordings (Extended Data Fig. 9b, c). In addition, tagged MD neurons showed a spatial localization that is predicted by their anatomy (Extended Data Fig. 9f, g).

To examine whether these selectivity patterns translate to effects on behaviour, we performed optical inactivation of MD_GRIK4_ neurons or their terminals in the PL. Both manipulations reproduced generic MD inactivation (Fig. 4e, Extended Data Fig. 9d), confirming that this specific neural opulation suppresses the PL when its inputs are uncertain due to conflict.

Given that MD suppression did not affect behaviour in trials in which cueing uncertainty was low, we reasoned that the PL can maintain these task inputs without requiring thalamic amplification. As such, we predicted that inactivation of MD_D2_ neurons (or their terminals in the PL) would have no effect on task performance (Supplementary Note 2). Although this prediction was validated for trials with low conflict, it resulted in performance improvement on trials with high conflict (Fig. 4f, Extended Data Fig. 9e). This finding raised the hypothesis that MD_D2_ neurons must be engaged during the cueing period, and under our current task conditions they would be amplifying prefrontal signals in a manner that is detrimental to behaviour. We tested this idea first by modifying our neural model to incorporate the hypothesized function of the two thalamic cell types (Fig. 4g), which reproduced the data on one end (Fig. 4h) and provided computational insight into the idea that MD_D2_-dependent amplification would increase the likelihood of non-preferred prefrontal inputs generating an erroneous choice (Extended Data Fig. 8c, d).

## MD_D2_ neurons are required when inputs are sparse

**Extended Data Fig. 10:**
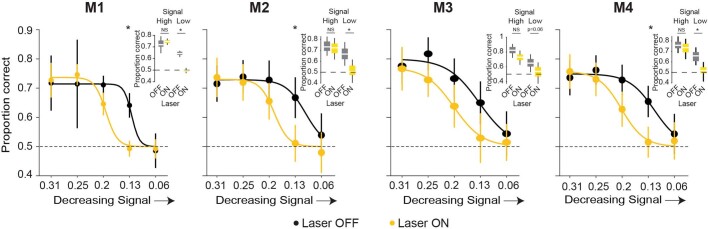
Full psychometric functions of individual mice in the sparseness task. Performance accuracy in the distributed cue task with input uncertainty due to cueing sparseness, separated by animals. For each animal, performance accuracy consistently diminishes with decreasing signal (black traces), while optical inhibition of PL→MD terminals (yellow traces) during the cueing period generally reduces performance more strongly on low signal trials than on high signal trials (M1: p = 0.644 (NS; relative signal = 0.25), *p = 0.0348 (relative signal = 0.13); M2: p = 0.676 (NS; relative signal = 0.25), *p = 0.0426 (relative signal = 0.13); M3: p = 0.139 (NS, relative signal = 0.25), p = 0.0604 (NS; relative signal = 0.13); M4: p = 0.343 (NS; relative signal = 0.25), **p = 0.0251 (relative signal = 0.13); chi-squared test). Inset in each panel highlights the inactivation effect on trials with high (0.25) and low (0.13) signal. All statistical tests are two-tailed. For inset box plots, boundaries, 25–75th percentiles; midline, median; whiskers, minimum–maximum. Data are presented as mean ± SEM Source data.

If MD_D2_ neurons were amplifying functional cortical connectivity underlying the generation of a choice signal, we sought to ascertain whether there are conditions under which eliminating this MD_D2_ neural function would be detrimental to performance. We reasoned that if the task uncertainty was not due to input conflict and instead due to input sparseness, this thalamic function may be required for optimal task performance. We first explored this conjecture in the model (Methods) and found it plausible (Extended Data Fig. 8e). Therefore, we designed a task in which we controlled input uncertainty by varying the degree of informative pulse sparseness within each sequence, rather than conflict (Fig. 4i). Because our earlier data indicated that MD inactivation was not required for such sequences when they included seven informative pulses, we varied their number between one and five in this new task design (Fig. 4j, Extended Data Fig. 10). We found that the MD is also causally required for this task in a manner that scales with uncertainty due to input sparseness (Fig. 4j). In other words, MD inactivation has a stronger effect on performance in trials with a low compared to a high input signal. Performing optical inactivation in cell-type specific Cre mice revealed that optimizing performance in this type of uncertainty condition was also segregated across these two thalamic populations: MD_D2_ neurons were required for performance in the low signal trials (Fig. 4l), whereas optical inhibition of MD_GRIK4_ neurons resulted in enhanced performance in both high and low signal trials under this task condition (Fig. 4k). Collectively, our experiments reveal a functional dissociation within MD–PFC loops in decision-making when inputs are uncertain. Specifically, MD_D2_ neurons that target disinhibitory VIP^+^ interneurons in the PL are required when task inputs are sparse (low signal), whereas MD_GRIK4_ neurons that target inhibitory PV neurons in the PL are required when inputs are dense but conflicting (high noise).

## Discussion

Although studies in humans have shown that MD thalamic activity tracks task input uncertainty, our ability to capture this process in mice has revealed, first, that these responses are heterogeneous at the single-cell level; and, second, that they effectively break down input uncertainty into two categories: low signal and high noise. Notably, these different neural signals are carried by two genetically distinct thalamic projections.

Our data may be relevant for identifying interventions in schizophrenia. Several studies have indicated a heightened susceptibility of patients to uncertainty during decision-making^24^, a process that may result in an unstable belief-updating process^25^. As such, examining how the MD–PFC network responds to different types of uncertainty and in the context of hierarchical decisions is likely to be of value (Supplementary Discussion). On the more mechanistic end, given that some of the leading aetiological hypotheses are related to PV interneurons (a target of MD_GRIK4_ neurons)^26,27^ and D2 receptors^28^ (a marker for MD_D2_ neurons), we are optimistic that our findings will provide key details to link recently discovered thalamocortical abnormalities^5,29^ to these classical ideas, opening up fresh avenues for therapeutic intervention.

## Methods

### Mice

A total of 94 mice were used in this study. Adult C57Bl/6 (wild-type) mice, of both sexes, aged 8–12 weeks old were purchased from Taconic Biosciences. GRIK4-cre, PV-cre, VIP-cre and SST-cre mice, of both sexes and aged between 8–12 weeks, were obtained from The Jackson Laboratory. D2-cre mice (GENSAT, line ER44), of both sexes and aged between 8 and 12 weeks, were a gift from M. Heiman. Cre mice were backcrossed to C57Bl/6 mice for at least six generations. All mice were kept in rooms with controlled temperature and ventilation (20–22 °C; 40–60% humidity) on a constant 12-h light–dark cycle. Mice were group housed with ad libidum access to food and water. All mouse experiments were performed according to the guidelines of the US National Institutes of Health and the Institutional Animal Care and Use Committee at the Massachusetts Institute of Technology.

### Viruses

For retrograde monosynaptic tracing, EnvA-RVdG expressing mCherry (titre: 1.9 × 10^11^ vp ml^−1^) was provided by I. Wickersham. Helper viruses AAV1-syn-FLEX-TA-TVA-GFP and AAV1-TREtight-B19G) for monosynaptic tracing were also provided by I. Wickersham (titre: 1.0 × 10^13^ vp ml^−1^). Retrograde AAV expressing Cre (AAVrg-hSyn-Cre-WPRE-hGH) was sourced from Addgene vector core (Addgene, lot 105553, titre: 7.0 × 10^12^ vp ml^−1^). For optogenetic manipulation experiments, AAV1-CamKIIa-SSFO-eYFP (titre: 1.0 × 10^13^ vp ml^−1^), AAV1-syn-ChR2-eYFP-Kv (titre: 4.6 × 10^12^ vp ml^−1^), AAV2-CamkII-eNPHR3.0-eYFP (titre: 1.5 × 10^12^ vp ml^−1^) and AAV2-EF1a-DIO-eNpHR3.0-eYFP (titre: 4.1 × 10^12^ vp ml^−1^) were sourced from UNC vector. AAV8-EF1a-DiO-iC++-eYFP (titre: 1.5 × 10^13^ vp ml^−1^) and AAV8-CamKIIa-iC++-eYFP (titre: 1.5 × 10^13^ vp ml^−1^) were sourced from the Stanford Vector core. mGRASP labelling studies were performed using viruses AAV2/8-CAG-JxON-pre-mGRASP (titre: 2.0 × 10^13^ vp ml^−1^) and AAV2/8-CAG-post-mGRASP-2A-dTomato (titre: 1.0 × 10^13^ vp ml^−1^) sourced from Neurophotonics, University of Laval. For our intersectional approach to label MD_D2_ neurons in wild-type mice we used an AAV-8- D2SP-Cre-P2A-mCherry (titre: 1.50 × 10^12^ vp ml^−1^) that drove mCherry and Cre expression under a D2-neuron-specific promoter. Simultaneous injections of another AAV-DJ hSyn Coff/Fon eYFP-WPREF (titre: 1.0 × 10^12^ vp ml^−1^) allowed expression of YFP in Cre-negative (CreOFF) neurons. For cell-type-specific MD→PL Kolmogorov–Smirnov labelling experiments, an AAV-DJ-hSyn-FLEX-mGFP-2A-Synaptophysin-mRuby (titre: 1.5 × 10^13^ vp ml^−1^) virus was used.

### Surgeries for anatomical tracing studies

Mice were first anaesthetized in an induction chamber receiving a continuous supply of oxygen and 5% isoflurane and then placed on a heating pad within a stereotaxic frame (Kopf Instruments). Throughout the surgery, anaesthesia was maintained through continuous delivery of 1–2% isoflurane via a nose cone at a rate of 1 l min^−1^ and analgesia was provided by dual subcutaneous injections of slow-release buprenorphine (0.1 mg kg^−1^) and Meloxicam (1 mg kg^−1^). The midline of the scalp was sectioned and retracted, and a small craniotomy was made over the target region. After levelling the head, a small burr hole was made over each target region using coordinates based on the mouse brain atlas of Paxinos and Franklin^30^. The coordinates are as follows (in mm from bregma): PL: antero-posterior (AP) 2.6, medio-lateral (ML) ±0.3, dorso-ventral (DV) −1.9; MD: AP −1.1, ML ±0.6, DV −3.0; A1: AP −2.92, ML ±4, DV −2.6; medial geniculate body (MGB), AP −3.0, ML ±2.05, DV −2.9 (from brain surface). For monosynaptic retrograde tracing experiments 300 nl of helper AAVs (1:1 mix of AAV1-syn-FLEX-TA-TVA-GFP and AAV1-TREtight-B19G) were injected into the PL of PV-cre, VIP-cre or SST-cre mice. Two weeks later, 100 nl of RVdG-mBFP2 (envA) was injected into the PL. Five days later the mice were euthanized to visualize monosynaptically labelled PL projection neurons in the MD (Fig. 2, Extended Data Fig. 3) and starter populations in the PL (Extended Data Fig. 3). To label cell-type-specific thalamocortical synapses, from MD neurons onto cortical PV^+^ and VIP^+^ interneurons with mGRASP (Extended Data Fig. 4), 75 nl of AAV2/8-CAG-JxON-pre-mGRASP (Cre-dependent) was injected into the MD and 200 nl of AAV2/8-CAG-post-mGRASP-2A-dTomato was injected into the PL of GRIK4-cre and D2-cre mice. Mice were given two weeks for expression of fluorescent proteins before being perfused as described in ‘Histology and immunohistochemistry’ below. To label MD→PL synaptic terminal densities across layers of the PL (Extended Data Fig. 4) we injected 75 nl of AAV-DJ-hSyn-FLEX-mGFP-2A-Synaptophysin-mRuby into the MD of GRIK4-cre and D2-cre mice. Mice were given two weeks for expression of fluorescent proteins before being perfused as described in ‘Histology and immunohistochemistry’ below.

Viruses were injected through a glass micropipette (Drummond Scientific) using a quintessential stereotactic injector (QSI, Stoelting) at a flow rate of 50 nl min^−1^ and given 10 min to spread after injection. After the injection micropipettes were slowly retracted followed by closure of the incision.

### Alternative strategy to target MD_D2_ neurons

Here we use a viral strategy to target D2^+^ neurons in the MD, independent of transgenic Cre lines. To this end, we injected an AAV with a short promoter that was previously established to express in D2 neurons only (AAV-8-D2SP-Cre-P2A-mCherry)^31^. This allowed us to examine neurons that are both D2^+^ (with a Cre-ON fluorophore) and D2^−^ through a simultaneous injection of another virus (AAV-DJ hSyn Coff/Fon eYFP-WPREF) that expresses only in the absence of Cre (Cre-OFF fluorophore^32^). Fourteen days after injection of a 1:1 mixture of the two viruses into the MD we found a substantial overlap between neurons that were D2^+^ with this approach and neurons that are D2^+^ in the Cre line as well as neurons that project to VIP interneurons on the basis of rabies tracing.

### Histology and immunohistochemistry

Mice were transcardially perfused with 30 ml of 0.1 M phosphate-buffered saline (PBS) followed by 20 ml of 4% paraformaldehyde (PFA) prepared in PBS. Brains were allowed to post-fix in the same fixative, overnight at 4 °C, then cryoprotected in 30% sucrose prepared in PBS for 24 h. Serial 50-µm-thick coronal sections were prepared using a Thermo HM550 cryotome. The GFP signal from the TVA helper constructs as well as the EYFP signal fused to opsins were enhanced with immunohistochemistry. In brief, sections were permeabilized and blocked in 10% bovine serum albumin (BSA, Sigma-Millipore) in PBS with 0.3% Triton X-100 (PBSTx) for 1 h. Then, sections were incubated overnight at 4 °C in primary chicken anti-GFP antibody (1:1,000, Aves Labs, GFP1011) prepared in PBSTx with 3% BSA. After two further washes, sections were incubated in an Alexa Fluor 488 goat anti-chicken secondary antibody (1:500, Thermo Fisher Scientific, A32931) for 2 h at room temperature, washed again and mounted for imaging. For mGRASP experiments, a similar protocol was followed to immunostain alternatePL sections (50 µm thick) from each brain for PL PV^+^ and VIP^+^ interneurons. We used rabbit anti-PV (1:1,000, Swant, PV-27) and rabbit anti-VIP (1:200, Immunostar, 20077) primary antibodies and an Alexa Fluor 647 donkey anti-rabbit secondary antibody (1:200, Thermo Fisher Scientific, A31573). GRIK4 protein was detected by an anti-rabbit primary GRIK4 antibody (1: 100, Alomone labs, AGC-041). For all viral injections, specificity of injection sites weas verified using virally expressed fluorescent proteins (GFP, EYFP, mCherry). Mice in which injection sites missed the target location were discarded from further analysis.

### In situ hybridization

Fresh-frozen brains from adult C57BL/6NJ mice (8–12 weeks) were sectioned at a thickness of 20 µm using a cryostat (Thermo Fisher Scientific). Sections were collected onto Superfrost Plus slides, immediately stored in a −20 °C freezer for 1 h for tissue adherence and subsequently transferred to a −80 °C freezer until staining. The D2 receptor mRNA signal was detected using the RNAscope fluorescent kit (Advanced Cell Diagnostics). Specifically, slides with sections corresponding to the MD were removed from the freezer, fixed with fresh and chilled 4% PFA for 15 min at 4 °C and then dehydrated using a series of ethanol solutions of increasing concentrations (5 min each, room temperature): once 50%, once 70% and twice 100%. Next, sections were treated with hydrogen peroxide for 10 min followed by Protease IV (Advanced Cell Diagnostics) at room temperature for 30 min. Hybridization was performed on a HybEZ (Advanced Cell Diagnostics) oven for 2 h at 40 °C using a mouse-specific D2 probe (Advanced Cell Diagnostics). After this, the slides were washed twice with a washing buffer (2 min each), then incubated with Hybridize Amp 1-FL for 30 min, Hybridize Amp 2-FL for 15 min and Hybridize Amp 3-FL for 30 min. Next, slides were incubated in horseradish peroxidase followed by TSA Plus Cyanine 3 fluorescent dye (1:750, Akoya Biosciences) for 30 min each at 40 °C. Next, HRP blocker was added for 10 min at 40 °C followed by counterstaining with DAPI for 30 s. The slides were washed twice with washing buffer (2 min each) and coverslips added using Prolong antifade mounting medium (Thermo Fisher Scientific). For negative controls the D2R probe was substituted with a probe against the *dapB* gene from the soil bacterium *Bacillus subtilis* while keeping all other steps the same.

### Image analysis

For monosynaptic input tracing experiments, images were acquired on a confocal microscope (LSM 710, Zeiss) with a 20×/0.80 numerical aperture objective (Zeiss) and analysed using Imaris Image analysis software (Imaris 9.3.2, Oxford Instruments). Images were manually overlaid with vectorized outlines from a modified version of the Reference atlas from the Allen Brain Atlas (Unified anatomical atlas)^33^ using anatomical landmarks as guides. Co-expression of GFP from the TVA-expressing helper virus and mBFP2 from the rabies virus were used to find bona fide starter neurons in the PL. Only those brains in which the starter neuron location was confined to the PL were processed for further analysis.

Monosynaptically labelled input cells, expressing mBFP2, were counted and their anatomical locations within the lateral MD recorded as follows. We measured the perpendicular distance of a candidate neuron from the lateral (medio-lateral distance axis) and ventral (dorso-ventral distance axis) boundaries of the MDl using the distance measure tool within Imaris. Their antero-posterior distance was measured from the anteriormost bregma location (AP −1.2 mm) where the MD is distinguishable into its three subdivisions—lateral, central and medial. The same method described above was used to image and record the anatomical location of MD_GRIK4_ and MD_D2_ neurons expressing mCherry in GRIK4-cre and D2-cre lines, respectively, as well as MD_D2SP_ neurons (Fig. 2, Extended Data Fig. 3).

These three distance measures (dorso-ventral, medio-lateral and antero-posterior distances) were used to perform a *k*-nearest neighbours (KNN) algorithm-based classification and cross-validation to examine anatomical separability of the prefrontal PV- and VIP-projecting MD neurons as well as the anatomical separability of MD_GRIK4_ and MD_D2_ neurons. In brief, each neuron is classified on the basis of a popularity vote of the identity of its five nearest neighbours, categorized as the most common identity among the five. The algorithm is repeated 100 times using 10-fold cross-validation. Neurons outside the 2.5% to 97.5% percentile, in any of the three distance axes, were excluded from further analysis as outliers.

As a second independent measure to validate the KNN-based classification, we performed representational similarity analysis. For each MD population (PV-projecting, VIP-projecting, MD_GRIK4_, MD_D2_ and MD_D2SP_), neuronal density is constructed along a three-dimensional (3D) space. The boundaries of the 3D space on each axis are placed at the minimum and maximum of location coordinates across all neurons. The 3D space is subsequently filled with evenly distributed nodes, with 10 each across the medio-lateral and dorso-ventral axis and 3 across antero-posterior axis (a total of 300 nodes), and the neuronal density is computed at each node. The representational similarity is computed as the Pearson correlation of the densities between different MD populations. When comparing within a population the comparison is performed across densities from 2 randomly separated halves, and the process is repeated 100 times.

To determine the laminar distribution of cell-type-specific MD terminal innervations, PL sections were imaged on a confocal microscope(LSM 710, Zeiss) with a 20×/0.80 numerical aperture objective (Zeiss). Multiple optical sections (1μ-m thickness) were imaged to cover the entire *z* axis of the section and reconstructed in 3D using Imaris. The acquired image was subdivided into 50-µm-wide bins starting from the pial surface and the volume of fluorescent signal, from the synaptically tagged GFP within a bin, was quantified normalized to the total volume of GFP fluorescence across all the bins. Laminar layers within were delineated using ‘unified anatomical atlas’ demarcations^33^.

For analysis of synapses labelled by mGRASP (Extended Data Fig. 4), PL sections were imaged using a confocal microscope (LSM 710, Zeiss) and 63×/1.40 numerical aperture objectives (Zeiss). Appropriate excitation wavelengths were used for different fluorescent protein markers: 488 nm for GFP (mGRASP-labelled synapses), 561 nm for TdTomato (post-mGRASP-labelled postsynaptic neurons) and 633 nm to detect anti PV or anti VIP immunohistochemistry fluorescent signal. Multiple optical sections (1-µm thickness) were imaged to cover the entire *z* axis of the section. Thereafter images were reconstructed in 3D and analysed using Imaris Image analysis software (Imaris 9.3.2, Oxford Instruments). Three-dimensional isosurfaces (smoothness, 0.2 mm; quality level, 5) were created for each PV or VIP neuron identified by the co-expression of the post mGRASP TdTomato signal and immunohistochemistry for PV or VIP. A mask was then created to isolate the fluorescent signals within and surrounding the cell body to eliminate fluorescent signals from outside the cell boundaries. For each masked cell, a second round of 3D isosurfaces were created (smoothness, 0.1 mm; quality level, 7) for the mGRASP signal. Care was taken to ensure that the entire mGRASP signal was covered by the isosurfaces created. The number of such isosurfaces created was used to quantify the number of synapses per cell.

A similar approach was used to quantify GRIK4 expression in MD_GRIK4_ and MD_D2_ neurons. In brief, after acquisition, the images were reconstructed in 3D and analysed using Imaris. Three-dimensional isosurfaces (smoothness, 0.2 mm; quality level, 5) were created for each MD_GRIK4_ or MD_D2_ neuron identified by reporter fluorescence. Subsequently the mean intensity of GRIK4 immunolabelled fluorescent signal within each isosurface is used to quantify GRIK4 expression in the corresponding neuron.

To quantify D2 receptor mRNA expression in the MD using in situ hybridization as described above, stained slides were imaged in an LSM710 laser-scanning confocal microscope at 40× magnification. The lateral MD region from each section was isolated using ImageJ and individual images were merged into a stack. Then a maximum intensity projection of the stack in the *z* plane was generated using the ‘stacks’ plug-in in ImageJ and visualized as a heat map using the ‘EzColocalization’ plug-in^34^.

### Multi-electrode array construction and implantation

Custom multi-electrode array scaffolds (drive bodies) were designed using 3D CAD software (SolidWorks) and printed in Accura 55 plastic (American Precision Prototyping) as described in previous studies^35^. Before implantation, each array scaffold was loaded with 16–24 independently movable micro-drives carrying 12.5-μm nichrome (California Fine Wire) tetrodes. Electrodes were pinned to custom-designed, 64- or 96-channel electrode interface boards (EIB, Sunstone Circuits) along with a common reference wire (A-M Systems). For combined optogenetic manipulations and electrophysiological recordings, optic fibres (Doric Lenses) were embedded above or adjacent (for fibres equipped with a 45-degree mirror tip) to the electrodes. For analgesia, mice were injected with slow-release buprenorphine (1 mg kg^−1^) before surgery. Then mice were deeply anaesthetized with 1% isofluorane and mounted on a stereotactic frame. The mouse head was shaved, and remaining hair removed with Nair. Body temperature was measured through a rectal probe and maintained using an electrical heating pad. An incision in the skin allowed access to the skull. An approximately 1.2 × 1.6-mm craniotomy was drilled centred at (in mm from bregma) AP 2, ML 0.6 for PL; at AP −1, ML 0.5 for MD; at AP −2.8, ML 4 for A1; and at AP −3.0, ML 2.0, DL 3.3 for MGB recordings. The dura was carefully removed, and the drive implant was lowered into the craniotomy using a stereotactic arm until the shortest tetrodes touched the cortical surface. Surgilube (Savage Laboratories) was applied around electrodes to guard against fixation through dental cement. Stainless steel screws were implanted into the skull to provide electrical and mechanical stability and the entire array was secured to the skull using dental cement. The skin was subsequently closed with Vetbond and the mouse was allowed to recover on a heating blanket.

### Head fixation recordings

Simultaneous recordings from MD and PL or MGB and A1 were conducted in a custom-built set-up. The head-fixation system consisted of a pair of custom 3D printed plastic fixation clamps (MakerBot Replicator) used to lock the implanted plastic crown at the base of the implant into place during recordings. These were fixed to an acrylic plastic frame which also supported a platform on which the mouse stood. The platform was composed of low-friction acrylic and was adjusted based on the height of the mouse and spring-loaded to minimize torque on the implant.

### Electrophysiological recordings

Signals from tetrodes (thalamic recordings) were acquired using a Neuralynx multiplexing digital recording system (Neuralynx) via a combination of 64- and 96-channel digital multiplexing head stages plugged to the 64–96 channel EIB of the implant. Signals from each electrode were amplified, filtered between 0.1 kHz and 9 kHz and digitized at 30 kHz. For thalamic recordings, tetrodes were lowered from the cortex into MD −2.8 to −3.2 mm DV and into the MGB −2.8 to −3.2 mm DV. For PL recordings, adjustments accounted for the change of depth of PL across the anterior-posterior axis. Thus, in anterior regions, unit recordings were obtained between-1.2 to −1.7 mm DV whereas for more posterior recordings electrodes were lowered −2 to −2.4 mm DV. For A1 unit recordings were obtained between −2.5 to −3.0 mm DV. Following acquisition, spike sorting was performed offline on the basis of relative spike amplitude and energy within electrode pairs using the MClust toolbox (http://redishlab.neuroscience.umn.edu/mclust/MClust.html).

### Identification of fast spiking and regular spiking cells

After initial spike sorting, PL units were divided into fast spiking (FS) and regular spiking (RS) according to waveform characteristics and spike rate as described previously^7^. Basic features of spike waveforms, including peak to trough time, half trough time, and trough depth, were measured for each unit across all spike waveforms. We also incorporated a measure of spike timing that has previously been used to identify FS neurons (spike rate)^36^. Recorded neurons were then separated using a clustering method for the four feature dimensions: (1) half trough time; (2) peak to trough time; (3) tough depth; and (4) spike rate. Clustering across the four dimensions were assessed using *k*-means clustering as described previously.

### Connectivity assay

To assess the effect of changes in thalamic excitability on cortical connection strength, we measured intra-cortical responses evoked by ChR2-mediated activation of the contralateral cortex for A1–MGB and PL–MD. Responses to either cortical stimulation alone (10 ms ChR2 activation to the contralateral cortex), thalamic activation alone (500 ms stabilized step function opsin (SSFO) activation in ipsilateral MGB or MD) or the combination were recorded in A1 and PL (50 interleaved trials per condition). For the combined condition, thalamic activation preceded cortical stimulation by 100 ms. To test the role of PL VIP neurons on MD-driven amplification of cortical connection strength, we also measured the responses of contralateral cortical stimulation alone, ipsilateral MD stimulation alone or combined stimulation with concurrent suppression of PL VIP neurons (1,000 ms NpHR3.0 activation) with an onset 500 ms before ChR2 activation).

For all cortical neurons, changes in baseline and evoked spike rates were assessed using peri-stimulus time histograms (PSTHs). PSTHs were computed using a 1 ms bin width for individual neurons in each recording session convolved with a Gaussian kernel (20 ms full width at half maximum) to create a spike density function (SDF). Evoked response through intracortical stimulation was measured as the baseline rate normalized delta between the maximum firing rate in a window 100 ms after ChR2 onset and the baseline rate measured over 500 ms before any laser stimulation. Proportional spike rate changes in the absence of contralateral cortical stimulation were calculated relative to the baseline rate.

### Behaviour

#### Set-up

Behavioural training and testing took place in custom-built enclosures as previously described^37^. All enclosures contained custom-designed operant ports, each equipped with an IR LED/IR phototransistor pair (Digikey) for nose-poke detection. An additional port for trial initiation was mounted on the floor 6 cm away from the ‘response ports’ located at the front of the chamber. Auditory cues and targets were presented with millisecond precision through a ceiling mounted speaker controlled by an RX8 Multi I/O processing system (Tucker-Davis Technologies). Visual stimuli were presented via two dimmable, white light emitting diodes (Mouser) mounted on each side of the initiation port. Two response ports were mounted at the angled front wall and a milk reward (10 μl evaporated milk) was directly delivered into the ports via a syringe pump (New Era Pump Systems) to reward correct choices. Access to the response ports was restricted by vertical sliding gates controlled through a servo motor (Tower Hobbies). The TDT Rx8 sound production system (Tucker Davis Technologies) was triggered through MATLAB (MathWorks), interfacing with a custom written software running on an Arduino Mega (Ivrea) for trial logic control. Across experiments, mice were randomly selected for training and all mice trained to criteria were included in testing. For optogenetic studies and physiological recording, mice were randomly selected from the overall cohort for inclusion in each type of manipulation or recording.

#### Training for the PL-dependent task

Training was largely similar to a previously described approach^7,37^. First, 10 µl of evaporated milk (reward) was delivered randomly to each reward port for shaping and reward habituation. Making response ports accessible signalled reward availability. Illumination of the LED at the spatially congruent side was used to establish the association with the visual targets on half of the trials while a similar presentation of a 100-ms tone cloud on the other half of the trials was used to build the association with the auditory target. An individual trial was terminated 15 s after reward collection, and a new trial became available 5 s later.

Second, mice learned to poke to receive a reward. All other parameters remained constant. An incorrect poke had no negative consequence. By the end of this training phase, all mice collected at least 20 rewards per 30-min session.

Third, mice were trained to initiate trials in which mice had to briefly (50 ms) break the infrared beam in the initiation port to trigger target stimulus presentation and render reward ports accessible. Trial rule (‘attend to vision’ or ‘attend to audition’) was indicated by 4 to 8 kHz low-pass (LP)-filtered white noise (vision) or 12 to 40 kHz high-pass (HP)-filtered white noise (audition) sound cues. Stimuli were presented in blocks of six trials consisting of single-modality stimulus presentation (no conflict). An incorrect response immediately rendered the response port inaccessible. Rewards were available for 15 s after correct poking, followed by a 5-s inter-trial interval (ITI). Incorrect poking was punished with a time-out, which consisted of a 30-s ITI. During an ITI, mice could not initiate new trials.

Fourth, conflict trials were introduced, in which auditory and visual targets were co-presented indicating reward at opposing locations. Trial types were presented in blocks of visual or auditory trials. The time that mice had to break the infrared barrier in the initiation port was continuously increased until it reached 0.8 s.

Fifth, trial availability and task rule were dissociated. Broadband white noise indicated trial availability, which prompted a mouse to initiate a trial. After successful initiation, the white noise was immediately replaced by either low-pass- or high-pass-filtered noise for 0.1 s to indicate the rule. This was followed by a delay period (variable, but for most experiments it was 0.4 s) before target stimuli presentation. All block structure was removed, and trial type was randomized. Mice were trained on this discrete cueing version of the task until mean performance plateaued and remained stable over 4–5 consecutive sessions (mean accuracy of 69 ± 3% correct). On a subset of trials, the two targets were shown on congruent sides to ensure that mice did not develop a pro-anti strategy for a single cue.

Mice were implanted with optic fibres in the PL and MD at this stage and retrained for testing with optogenetic manipulation (described below) for experiments involving a single HP or LP cueing pulse (Extended Data Fig. 5).

Sixth, single HP or LP pulses were replaced by sequences of several 50-ms-long pulses of either HP or LP, separated by a 25-ms gap of silence. In parallel, snout fixation duration was increased until a total of 16 pulses could fit within the cueing period (1,200 ms). Finally, unlike the single-pulse version of the task, the noise-free delay between the end of the cueing pulses and the presentation of choice targets was intentionally kept below 250 ms to focus our study on uncertainty in sensory inputs. Once the mice performed on these ‘pure’ sequences equivalent to the single-pulse trials, input uncertainty trial types were introduced in which the evidence varied for attend to vision versus attend to audition. Conflict-driven input uncertainty trials were generated by incorporating different mixtures of HP, LP, and broadband white noise (conflict mediated uncertainty). Out of the 16 pulses, only 9 conveyed rule information (either HP or LP). The remaining seven pulses consisted of broadband white noise. Low-signal-driven input uncertainty trials only contained one type of meaningful pulses (either HP or LP) embedded in broadband white noise pulses. Out of the 16 pulses, only 1 to 5 pulses were meaningful to make those cueing sequences sparse in signal. Mice were required to select the appropriate target stimulus based on the rule with the highest number of corresponding pulses on a trial-by-trial basis. Trial types were presented in random order.

#### Training for the PL-independent task

The first two training steps were similar to the PL-dependent 2AFC task except the target modality was restricted to the visual domain where an LED was illuminated for 10 ms at a spatially congruent side to indicate rewarded response port. In the next stage of training mice were trained to initiate trials in which they had to briefly (50 ms) break the infrared beam in the initiation port to trigger target stimulus presentation and render reward ports accessible. Target stimuli were presented in blocks of six trials consisting of single-modality stimulus presentation (no conflict). An incorrectresponse immediately rendered the response port inaccessible. Rewards were available for 15 s after correct poking, followed by a 5-s ITI. Incorrect poking was punished with a time-out, which consisted of a 30-s ITI. During an ITI, mice could not initiate new trials. On the final stage of the task trial availability and target presentation were dissociated. Broadband white noise indicated trial availability, which prompted a mouse to initiate a trial. After successful initiation, the white noise was immediately replaced by illumination of a LED light on the left or right to indicate the response port where reward was available. All block structure was removed, and trial type was randomized. Mice were trained on this version of the task until performance plateaued and remained stable over 4–5 consecutive sessions.

#### Optogenetic manipulation

We used a dual wavelength optical silencing method to independently suppress neurons in the PL and MD. Specifically, we virally expressed halorhodopsin (AAV2-CamkII-eNPHR3.0-eYFP) in the PL and a Cre-dependent (in GRIK4-cre and D2-cre mice; AAV8-EF1a-DiO-iC++-eYFP) or Cre-independent (in wild type mice; AAV8-CamKIIa-iC++-eYFP) inhibitory channelrhodopsin iC++ in the MD. As the peak spectrum of NpHR3.0 is red-shifted (peak around 550 nm), we could independently inactivate both populations or their terminals in either structure, through implanted optic fibres, using a 473-nm and a 556-nm laser (OptoEngine) to activate iC++ and NpHR3.0 respectively. For all optogenetic experiments (Figs. 3, 4,Extended Data Figs. 6, 7, 9, 10), optogenetic trials were randomly interleaved among other trial types and investigators were blinded to trial type; longitudinal comparisons were then used within individuals between trial types. This is true except for experiments in which the role of MD in task engagement was evaluated (Extended Data Fig. 5), or inthe optotagging experiments (Fig. 4). In the former experiments, optogenetic inactivation of the MD was done on trial number 1 to 30 of the session (Extended Data Fig. 5). Laser duration varied depending on the trial type between 100 ms (during single-pulse cueing period; Extended Data Fig. 5), 400 ms (single-pulse delay period; Extended Data Fig. 5) and 1,200 ms (entire cueing period of a 16-pulse cueingsequence). In the latter experiments, optogenetic tagging was performed after the behaviour session (see below). During a session, only one condition was tested with optogenetic manipulation.

#### Firing rate analysis

For all thalamic and cortical neurons, changes in spike rates associated with task performance were assessed using PSTHs. PSTHs were computed using a 1 ms bin width for individual neurons in each recording session convolved with a Gaussian kernel (20 ms full width at half maximum) to create an SDF. Proportional firing rate change was calculated relative to a 500-ms-long baseline before event onset. Notably, all task-related rasters and PSTHs (and neural analysis such as decoding analysis) are aligned to cue onset (*t* = 0).

### Classification of thalamic neurons into conflict-preferring versus conflict-non-preferring

Conflict-preferring and conflict-non-preferring neurons were identified using the area under receiver operating characteristics (auROC) method. In brief, auROC provides an aggregate measure of the association between single-trial firing rates and trial type, across levels of response. For each neuron, the proportional response for each trial was computed over the 300–1,200 ms window after cue onset (the beginning of the cueing period, when the conflict signal had just began to emerge, was omitted). The fraction of trials for which the proportional response exceeds a threshold, as a function of varying threshold, was computedover two trial types (for example, low conflict trials and high conflict trials). The ROC curves are pairs of fractions for the two trial types (*f*_1_, *f*_2_) over each shared threshold value, plotted with one trial type over one axis. As such, the ROC curve goes from (0,0) (when the threshold is higher than the response in all trials) to (1,1) (when the threshold is lower than the response in all trials). The auROC computes the area below the ROC curve between (0,0) and (1,1). All neurons from the population of interest were pooled together and their auROC was computed as above. Neurons with auROC significantly above 0.5 (that is, > 1.5 standard deviation (SD)) for high versus low conflict trials are defined as conflict-preferring. Neurons with auROC significantly above 0.5 (that is, > 1.5 SD) for low versus high conflict trials are defined as conflict-non-preferring.

### Decoding analysis

Trial-by-trial classification analysis was performed using a support vector machine (SVM) implemented through LIBSVM and MATLAB (Mathworks) neural decoding toolbox^38^, similar to previously reported^39^. To perform decoding on cue, choice or conflict, the firing rates of neurons on each trial from the entire population (pooled across sessions) were first smoothed using a Gaussian filter of 20 ms width. The SVM classifier with a Gaussian radial basis function kernel was then trained on (randomly selected) half of the data and tested on the other half of the data, with a sliding window of 300 ms and time step of 100 ms. The classes were balanced during training, such that an equal number of trials were (randomly) selected for each class. This classifier works by first constructing an optimal hyperplane based on labelled training data and then generating predictions of the labels on testing data. Accuracy of the decoding was assessed by comparing the predicted labels to the actual labels. Classification accuracy was also quantified by computing the mutual information via the following equation:

$${\rm{MI}}=\mathop{\sum }\limits_{i=1}^{s}\mathop{\sum }\limits_{j=1}^{s}{p}_{ij}\,\log \,\frac{{p}_{ij}}{{p}_{i}{p}_{j}}$$

where *p*_*ij*_ is the probability of observing label *i* (cue, choice, or conflict) given that the original label is *j*. This classification process was repeated 100 times to obtain and accurately estimate the error of the classification accuracy.

To analyse the separability of conflict and choice information in MD and PL, 50 of the most conflict-selective MD neurons, and 50 of the most choice-selective PL putative excitatory neurons, are pooled. Decoding is performed as described above, and the maximum classification accuracy is computed.

### Optogenetic tagging and identification of cell-type-specific MD neurons

GRIK4-cre and D2-cre mice trained on the cueing uncertainty version of the attention control task were injected with AAV2-EF1a-DIO-eNpHR3.0-eYFP in the MD and implanted with multi electrode arrays and optic fibres targeted to the MD. After every behaviour session, and in a separate box outside of task context, each mouse received 50 trials of 10-ms-long pulses of eNpHR3.0 stimulation. Three features of a the response of an MD neuron to eNpHR3.0 stimulation were measured for each neuron in a 50-ms window after eNpHR3.0 stimulation: (1) change in mean proportional spike rates; (2) fraction of trials with spike rate suppression; and (3) recovery half-time (Extended Data Fig. 9a). Tagged neurons were identified using *k*-means clustering across the three dimensions. Optotagged clusters of MD_GRIK4_ or MD_D2_ neurons so identified demonstrated a strong decrease in proportional spike rates and high fraction of trials with rate suppression. Subsequently, the tagged MD_GRIK4_ or MD_D2_ neurons were classified into conflict-preferring versus conflict-non-preferring from the responses recorded in the preceding behaviour session.

### Neural model for decision-making circuit

To study how MD may optimize PL computation in generating choice signal under input conflict, we constructed a neural mean field model (reduced form of a spiking circuit model) of the PL circuit executing a 2AFC decision-making task^40^. Whereas a spiking circuit model describes the temporal evolution of hundreds or thousands of neural units (under a defined circuit architecture), a mean-field model averages over homogeneous populations, smearing over interactions and resulting in a low-dimensional system with key dynamics of interest. Similar models were used in the literature to capture key features of human and primate behavioural and neural data^39^. Variants of the model regime had also shed light on the decision-making neural circuitry in mice^41^.

Specifically, our model (custom Python code) described two excitatory populations within the PL that received inputs corresponding to high-pass and low-pass pulses respectively, and the outputs of which would be read out to form the attentional choices. Each excitatory population had recurrent connections onto itself that allowed integration of the input pulses. The two populations also project to an inhibitory population that symmetrically suppresses both populations, resulting in competition between the two populations. We also incorporated MD→PL projections into the model as constrained by experimental data. We considered two different implementations of the MD module. In the first implementation (Extended Data Fig. 7o), MD dynamically computes cueing conflict to activate the PL inhibitory population and suppress both PL excitatory populations accordingly. The second implementation incorporated the two thalamic cell types, with MD_GRIK4_ dynamically activated under cueing conflict to suppress PL, whereas MD_D2_ was conflict-suppressed and amplified recurrence in PL.

The mean-field model described the temporal evolution of NMDA receptor (NMDA-R) gating variables of the two excitatory populations (*S*_1_, *S*_2_), which were also the decision variable representing the integrated evidence for the two choices. The model also included firing rates and other synaptic gating variables of the two populations. However, they were treated as steady states owing to their much shorter timescales than NMDA-R gating variables.

The two NMDA-R gating variables evolved according to:

1 $$\frac{{\rm{d}}{S}_{i}}{{\rm{d}}t}=-\frac{{S}_{i}}{{\tau }_{{\rm{NMDA}}}}+(1-{S}_{i})\gamma {r}_{i}$$

for *i* = 1,2. *τ*_NMDA_ = 100 ms and *γ* = 0.641 were the synaptic time constant and saturation factor for NMDA-R. *r*_1_, *r*_2_ were the firing rates of the two excitatory populations. These rates were computed from the transfer function based on the total input currents *I*_1_, *I*_2_. The input currents:

2 $${I}_{1}={\alpha }_{1}{S}_{1}+{\alpha }_{2}{S}_{2}+{\beta }_{1}{r}_{1}+{\beta }_{2}{r}_{2}+{I}_{1}^{{\rm{ext}}}$$

3 $${I}_{2}={\alpha }_{1}{S}_{2}+{\alpha }_{2}{S}_{1}+{\beta }_{1}{r}_{2}+{\beta }_{2}{r}_{1}+{I}_{2}^{{\rm{ext}}}$$

arose from the NMDA-Rs of the same population (for example, *α*_1_*S*_1_ in equation (1)) and competing population (for example, *α*_2_*S*_2_ in equation (2)), the AMPA receptor gating variables of the same population (for example, *β*_1_*r*_1_ in equation (2)) and competing population (for example, *β*_2_*r*_2_ in equation (2)), and external inputs (for example, ${I}_{1}^{{\rm{ext}}}$ in equation (2)). GABA receptor gating variables were also expressed in *α*_*i*_ and *β*_*i*_ to account for lateral inhibition. The synaptic parameter values are *α*_1_ = 0.164 nA, *α*_2_ = −0.022 nA, *β*_1_ = 9.9 × 10^−4^ nC, *β*_2_ = −6.5 × 10^−5^ nC. The external input ${I}_{1,2}^{{\rm{ext}}}$ is due to a constant but noisy input ${I}_{1,2}^{\eta }$, and a stimulus input ${I}_{1,2}^{{\rm{stim}}}$ $({I}_{1,2}^{{\rm{ext}}}={I}_{1,2}^{\eta }+{I}_{1,2}^{{\rm{stim}}})$ . ${I}_{1,2}^{\eta }$ is described by an Orntein-Ulhenbeck process with mean *I*_OU_ = 0.350 nA, noise *σ*_OU_ = 0.015 nA, and time constant *τ*_OU_ = 2 ms. ${I}_{1,2}^{{\rm{stim}}}$ = 0.017 nA under the presence of favoured input pulses, but 0 otherwise. Using change of variables ${x}_{1}={\alpha }_{1}{S}_{1}+{\alpha }_{2}{S}_{2}+{I}_{1}^{{\rm{ext}}},$ ${x}_{2}={\alpha }_{1}{S}_{2}+{\alpha }_{2}{S}_{1}+{I}_{2}^{{\rm{ext}}}$, the transfer function can be written as

4 $${r}_{1}=\frac{a{x}_{1}-f({x}_{2})-b}{1-\exp [-d(a{x}_{1}-f({x}_{2})-b)]}$$

5 $${r}_{2}=\frac{a{x}_{2}-f({x}_{1})-b}{1-\exp [-d(a{x}_{2}-f({x}_{1})-b)]}$$

where *a*, *b*, *d* were constants that depended on *β*_1_, and *f* was a function of *x*_*i*_ that depended on *β*_2_. The expression of ${\alpha }_{i},{\beta }_{i},a,b,d,f,{I}_{i}^{{\rm{ext}}}$ are detailed in a previous study^40^, but in brief, the transfer function results in a smooth and thresholded input–output response (Extended Data Fig. 8c, bottom). A choice was selected at the end of stimulus presentation, based on the population with higher decision variable (*S*_1_, *S*_2_). Stimulus inputs in general drove categorical, winner-take-all competitions such that the two decision variables were largely separated (with the loser decision variable near 0; Extended Data Fig. 8).

In the model with a generic MD (Extended Data Fig. 7o) inactivation, the effect of MD was incorporated as inhibitory inputs to the two PL populations in the presence of conflict $({I}_{1,2}^{{\rm{ext}}}={I}_{1,2}^{\eta }+{I}_{1,2}^{{\rm{stim}}}+{I}^{{\rm{MD}}})$. Conflict was dynamically computed by considering the current pulse and the last non-white-noise pulse (that is, if one pulse was HP and the other LP), although other implementations of conflict computation yielded consistent results. In addition, a baseline suppression to PL was added to dissociate the effects of MD inactivation versus MD deafferentation (Extended Data Fig. 7p, r) (*I*_MD_ = −0.1 nA under conflict, = −0.01 nA without conflict). In particular, MD inactivation removed all effect of MD, whereas the baseline suppression to PL remained under optical inhibition of PL→MD terminals.

In the model with two MD cell types (Fig. 4g), the effect of MD_GRIK4_ was similarly incorporated similarly as inhibitory inputs to the PL in the presence of conflict $({I}_{1,2}^{{\rm{ext}}}={I}_{1,2}^{\eta }+{I}_{1,2}^{{\rm{stim}}}+{I}^{{\rm{GRIK4}}})$ . However, the baseline suppression was removed for simplicity (*I*_GRIK4_ = −0.1 nA under conflict, 0 without conflict) considering similar effects of MD inactivation and optical inhibition of PL→MD terminals in the previous model. The effect of MD_D2_ was incorporated as an augmentation to the recurrent synaptic connections (8% increase to *β*_1_ and *β*_2_, equations (2) and (3)), resulting in a gain increase of the transfer function (equations (4) and (5); Extended Data Fig. 8c, bottom). In the models without MD_GRIK4_ or MD_D2_ (Fig. 4h), the corresponding module was removed. Finally, a slightly altered circuit model was used to demonstrate the viability that MD_D2_ may contribute to decision-making under input uncertainty due to cueing sparseness (Fig. 4j, Extended Data Fig. 8e). We reduced *I*_OU_ to slow down the rate for which decision variables approach attractor states. This corresponded to a slower integration process, allowing the model circuit to accumulate sparse evidence distributed across the cueing period, early or late. We note that this altered model was only used to generate example traces (Extended Data Fig. 8e) and was not used in any analysis.

### Regression analysis

Regression analysis was used to ensure mice used the entire cue sequence to inform their choice behaviour. In particular, a logistic regression model on choice (correct or error) was performed with the evidence in the first (early) and second (late) half of the cue sequence as regressors:

6 $$\mathrm{ln}\left(\frac{P}{1-P}\right)={\beta }_{0}+{\beta }_{e}|\mathop{\sum }\limits_{i=1}^{8}{C}_{i}|+{\beta }_{l}|\mathop{\sum }\limits_{i=9}^{16}{C}_{i}|,$$

where *P* is the probability to be correct, *C*_*i*_ is the *i*th pulse in the trial (= 1 for a low-pass pulse, = −1 for a high-pass pulse, = 0 for a white noise pulse), *β*_0_ is the bias term, and *β*_e_ and *β*_l_ reflect the degree the magnitude of momentary cues in the early and late half, respectively, contribute to animal choice behaviour.

### Statistical analysis

Statistical analysis was performed in MATLAB (Mathworks) and GraphPad Prism software (v.8.0, Prism). We did not assume normality in the distribution of our datasets and hence used two-sided non-parametric statistics to test for significance. For each statement of statistical difference included in the manuscript, a corresponding statistical comparison was performed, as mentioned in the figure legends. In brief, we used a Mann-Whitney *U* test for all comparisons between two groups comprising independent samples and a Wilcoxon signed-rank test when the samples were dependent. For comparison of cumulative distributions, the Kolmogorov–Smirnov test was used. For comparisons of observed proportions of binary (categorical) variables, we used a binomial test to compare to chance, and a chi-squared test to compare across two groups. For comparisons of decoding accuracies, we used permutation tests, rerunning the decoding analysis with shuffled trial labels, computing the fraction of trials exceeding the reported value. When comparing across conditions (laser off versus laser on), the shuffling is performed on neurons across conditions. For logistic regression, a two-sided Student’s *t*-test was used, as part of the output of MATLAB function glmfit. All *P* values are listed in the figure legends. Values are expressed as medians ± 95% range in box-and-whisker plots and mean ± s.e.m. for bar graphs.

#### Power analysis

For behavioural studies, power analyses were performed to determine the number of mice needed to establish an effect. Specifically, the MATLAB function sampsizepwr was used to estimate the number of mice. For the single-cue tasks, we performed a priori power analysis based on previously published data of the same task with MD manipulation^7,20^. The expected value and standard deviation of the null hypothesis (that optical manipulation has no effect), respectively, were 0.64 and 0.025, and the expected value of the alternative hypothesis (that optical manipulation abolishes performance) is 0.5, resulting in an effect size of Cohen’s *d* = 5.6. With a significance value of 0.05 and a power of 0.7, we estimated a number of 3 mice to be appropriate. We used 3–4 mice across experiments. Number of mice in each panel: Extended Data Fig. 5i, l: 4 mice; rest of Extended Data Fig. 5d–n: 3 mice.

For the conflict and sparseness tasks, we assumed similar variability in the data and effect size, thus resulting in the same estimated number of 3 mice. However, to be cautious with variability of the effect size we collected data from 4–6 mice for distinct optical manipulation experiments. Number of mice in each panel: Fig 3, Extended Data Fig. 7i: 5 mice; Fig. 4, Extended Data Fig. 9d, e: 4 mice of each genotype; Extended Data Figs. 6a–c, 7j,q: 6 mice. Also see Supplementary Table 1.

### Reporting summary

Further information on research design is available in the Nature Research Reporting Summary linked to this paper.

## Online content

Any methods, additional references, Nature Research reporting summaries, source data, extended data, supplementary information, acknowledgements, peer review information; details of author contributions and competing interests; and statements of data and code availability are available at 10.1038/s41586-021-04056-3.

## Supplementary information

Supplementary Information This file contains Supplementary Table 1, a Supplementary Introduction, two Supplementary Notes, a Supplementary Discussion, and Supplementary References.

Reporting Summary

## Data Availability

The data that support the findings of this study are available from the corresponding author upon reasonable request. Source data are provided with this paper.

## Source data

Source Data Fig. 1
Source Data Fig. 2
Source Data Fig. 3
Source Data Fig. 4
Source Data Extended Data Fig. 1
Source Data Extended Data Fig. 3
Source Data Extended Data Fig. 4
Source Data Extended Data Fig. 5
Source Data Extended Data Fig. 6
Source Data Extended Data Fig. 7
Source Data Extended Data Fig. 9
Source Data Extended Data Fig. 10

## Code availability

Custom codes for analysis and modelling were written in MATLAB and are available from the corresponding author upon request.
