# Supplementary material for: Thalamic circuits for independent control of prefrontal signal and noise
Source: Nature. 2021 Oct 6;600(7887):100–4. doi: 10.1038/s41586-021-04056-3 (PMC8636261; doi:10.1038/s41586-021-04056-3)
Supplement: Supplementary file 1 — This file contains Supplementary Table 1, a Supplementary Introduction, two Supplementary Notes, a Supplementary Discussion, and Supplementary References. [file 41586_2021_4056_MOESM1_ESM.pdf]

nature portfolio

https://doi.org/10.1038/s41586-021-04056-3

Supplementary information

Thalamic circuits for independent control of prefrontal signal and noise

In the format provided by the authors and unedited

Supplemental Table 1: Summary of contribution of individual animal to each figure

Behavior

Figure	Panel	Experiment	Animal ID (line)
3	b, e	Distributed cue task with conflict (PL/MD inactivation)	WT11, WT19, WT32, M2-5, M4-3 (C57/Bl6)
4	e	Distributed cue task with conflict (MD_{Grik4} inactivation)	GC4, GC7, GC10, GC12 (Grik4-cre)
	f	Distributed cue task with conflict (MD_{D2} inactivation)	D2C9, D2C11, D2C17, D2C20 (D2-cre)
	j	Distributed cue task with sparseness (PL->MD terminal suppression)	GC4, GC20 (Grik4-cre),<br>D2C9, D2C16 (D2-cre)
	k	Distributed cue task with sparseness (MD_{Grik4} inactivation)	GC4, GC7, GC10, GC12 (Grik4-cre)
	l	Distributed cue task with sparseness (MD_{D2} inactivation)	D2C9, D2C11, D2C17, D2C20 (D2-cre)
EDF5	c-f, i, l	PL dependent and idependent (visual detection) task (MD inactivation)	WT11, WT19, WT32, M4-3 (C57/Bl6)
	g, k, n	PL dependent (visual detection) task (MD_{D2} inactivation)	D2C2, D2C9, D2C17 (D2-cre)
	h, j, m	PL dependent (visual detection) task (MD_{Grik4} inactivation)	GC4, GC6, GC7 (Grik4-cre)
EDF6	a	Distributed cue task with conflict; valid or invalid target only	GC10, GC11, GC13 (Grik4-cre),<br>D2C15, D2C21, D2C22 (D2-cre)
	b	Distributed cue task with conflict; uninformative trials	WT11, WT19, M2-5, M4-3 (C57/Bl6),<br>SST1 (SST-cre), VIP2 (VIP-cre)
	c	Distributed cue task with conflict; regression model	WT11, WT19, WT32, M2-5, M4-3, S3 (C57/Bl6)
	d	Distributed cue task with conflict (PL/MD inactivation)	WT11, WT19, WT32, M2-5, M4-3 (C57/Bl6)
EDF7	i	Distributed cue task with conflict (PL/MD inactivation, intensity matched)	WT11, WT19, WT32, M2-5, M4-3 (C57/Bl6)
	j, q	Distributed cue task with conflict (PL->MD terminal suppression)	WT11, WT19, WT32, M2-5, M4-3, S3 (C57/Bl6)
	n	Distributed cue task with conflict (PL/MD inactivation)	WT11, WT19, WT32, M2-5, M4-3 (C57/Bl6)
EDF9	d	Distributed cue task with sparseness (MD_{Grik4}->PL inactivation)	GC4, GC7, GC10, GC12 (Grik4-cre)
	e	Distributed cue task with sparseness (MD_{D2}->PL inactivation)	D2C9, D2C11, D2C17, D2C20 (D2-cre)
EDF10	N/A	Distributed cue task with sparseness (PL->MD terminal suppression)	GC4, GC20 (Grik4-cre),<br>D2C9, D2C16 (D2-cre)

Electrophysiology

Figure	Panel	Experiment	Animal ID (line)
1	c, e, f	MD-PL connectivity assay	VIP4, VIP6, VIP7, VIP8 (VIP-cre)
2	m, n	MD-PL connectivity assay	GC1, GC33, GC34 (Grik4-cre), D2C31, D2C32, D2C3 (D2-cre)
3	d, g	PL & MD recordings in distributed cue task with conflict (PL/MD inactivation)	WT19, WT32, M4-3 (C57/Bl6), GC10, GC11 (Grik4-cre), D2C10, D2C21 (D2-cre)
4	b	MD recordings in distributed cue task with conflict (MD_{Grik4} optotagging experiments)	GC10, GC11, GC13 (Grik4-cre)
	d	MD recordings in distributed cue task with conflict (MD_{D2} optotagging experiments)	D2C10, D2C15, D2C21 (D2-cre)
EDF1	c	MD-PL connectivity assay	VIP4, VIP6, VIP7, VIP8 (VIP-cre)
	f	MGB-A1 connectivity assay	MGBA1_1, MGBA1_2, MGBA1_3 (C57/Bl6)
	g-k	MD-PL connectivity assay	VIP4, VIP6, VIP7, VIP8 (VIP-cre)
	g	MGB-A1 connectivity assay	MGBA1_1, MGBA1_2, MGBA1_3 (C57/Bl6)
EDF3	i	MD-PL connectivity assay	GC1, GC33, GC34 (Grik4-cre), D2C31, D2C32, D2C3 (D2-cre)
EDF7	b, c, e-h	PL & MD recordings in distributed cue task with conflict	WT19, WT32, M4-3 (C57/Bl6), GC10, GC11 (Grik4-cre), D2C10, D2C21 (D2-cre)
	k, l	PL & MD recordings in distributed cue task with conflict (PL->MD terminal suppression)	WT19, WT32, S3 (C57/Bl6)
	m	PL & MD recordings in distributed cue task with conflict (PL/MD inactivation)	WT19, WT32, M4-3 (C57/Bl6), GC10, GC11 (Grik4-cre), D2C10, D2C21 (D2-cre)
EDF9	b	MD recordings in distributed cue task with conflict (MD_{Grik4} optotagging experiments)	GC10, GC11, GC13 (Grik4-cre)
	c	MD recordings in distributed cue task with conflict (MD_{D2} optotagging experiments)	D2C10, D2C15, D2C21 (D2-cre)

Supplemental Introduction:

In decision making, the ability to estimate uncertainty is critical for optimizing outcomes[1]. Uncertainty can be about sensory inputs (input uncertainty[2]), their mapping onto internal/behavioral variables (rule uncertainty[3]), or their likelihood of predicting reward (outcome uncertainty[4]). Input uncertainty is mostly studied in sensory systems[5,6] but executive systems are also confronted with uncertain inputs that need to be turned into discrete control signals[7]. For example, at an event where multiple languages are spoken, it may take time to decide whether spoken words belong to one of two closely related languages, a form of uncertainty that may prohibit one from joining their preferred conversation. Conversely, uncertainty may be due to silence. In either scenario, resolving uncertainty is a prerequisite for directing attention, which is thought to involve the prefrontal cortex (PFC)[8]. Prefrontal dysfunction is found in disorders like schizophrenia, where patients may fail to efficiently and optimally deploy cognitive resources[9].

Recent studies have shown that the mediodorsal thalamus (MD) is a critical partner for the PFC in generating attentional signals, and cognitive control more broadly[10–12]. While earlier work in animals has shown that the MD enhances task relevant prefrontal activity patterns[13,14] and suppresses task irrelevant ones[15–17], recent studies in humans show that MD engagement in decision making scales with the degree of input uncertainty[18,19]. In this study, using mice, we found that the MD contains two genetically distinct cell types that play complementary roles in decision-making under uncertainty. These circuits act through inhibitory and disinhibitory prefrontal circuits, directly connecting prefrontal excitation/inhibition balance to decision making computations. Our work may provide an entry point for correcting decision-making abnormalities in schizophrenia where MD-PFC interactions are perturbed[20].

Supplemental Note 1 :

Identified MD cell types are differentially engaged in maintaining and switching attentional control

Previous studies have indicated two roles for the MD thalamus in PL-dependent tasks; enhancement of activity required for maintaining working memory and executive control signals[14,21,22], and suppression of activity required for task switching[23,24]. A clear case for this

functional segregation comes from an attentional control task that was previously developed and validated[14]. Briefly, in this task a freely behaving mouse selects between a visual and auditory target whose spatial locations are pseudorandomized (Extended Data Fig. 5a). On each trial, the selection process is guided by a 100 ms-long learned cue; either a high-pass (HP) or low-pass (LP) filtered white noise pulse, which correspond to one of two rules – *attend to audition* and *attend to vision* respectively. A mouse is required to hold the presented cue in mind over a delay prior to the simultaneous presentation of the two targets. The targets correspond to the spatial location of the reward being delivered through a right or left reward port (e.g. left LED flash on an attend to vision trial or an upsweep on an attend to audition trial signaled a response on the left reward port). Mice perform at chance level when the PL is optogenetically suppressed either during the cueing or delay periods of the task[14]. In contrast, when an animal achieves stable performance on the task, MD inactivation on single interleaved trials is only disruptive when delivered during the delay period[14]. However, MD inactivation during the cueing period has a detrimental effect when the cues are switched, and the MD exhibits unique electrophysiological signatures associated with prefrontal suppression[24]. These findings give rise to the notion that MD-dependent PL suppression is required for setting up the appropriate task-relevant connection patterns[24] which MD-dependent PL activation can later work to maintain[14,22].

Could these two functions be subsumed by the two identified MD cell types? To test this idea, we trained D2-cre and GRIK4-cre mice on the attentional control task described above (Extended Data Fig. 5a). Interestingly, we found that animals required several trials to achieve stable performance on this task, a quality that was not associated with a visual detection task that we previously showed was not PL dependent (Extended Data Fig. 5b-d). Therefore, we hypothesized that this initial 're-learning' requirement is the consequence of the PL needing to be configured for the attentional control task, and that this process may be MD dependent. Indeed, cue-specific MD suppression during this 're-learning' period resulted in animals taking longer to become engaged in the task (Extended Data Fig. 5e, i). Equivalent MD suppression in the PL-independent, visual detection, task had no effect on trials taken to get task engaged (Extended Data Fig. 5f). Importantly, we found that MD_{GRIK4} but not MD_{D2}, suppression resulted in a similar behavioral effect (Extended Data Fig. 5g-k). In contrast, MD_{D2} but not MD_{GRIK4} inactivation reproduced the total MD inactivation effects on behavior when delivered during the delay period of the task (Extended Data Fig. 5l-n). These experiments confirmed that the functional segregation of the

identified thalamic cell types have distinct behavioral effects on our previously established PL-dependent attention control task.

Supplemental Note 2:

Effect of MD activation across different time periods in the attention control task:

MD inactivation in the single cue task (original version):

1. In the original version of the task, where a single cue is delivered, MD inactivation during the cueing period has minimal impact on behavior in the steady state (i.e. when the animal established asymptotic performance within a session). (see Panels 2h,i in Schmitt et al., and 3e in Rikhye et al.)[14,24]

2. Under the conditions above, MD inactivation during the delay period diminishes task performance (See panels 2h, Extended Data Figure 6 in Schmitt et al.)[14]

3. Cue-specific MD inactivation diminishes task performance in the first few trials of task switching (See panels 3e-g, Supplementary Fig. 11 in Rikhye et al.)[24], which we now demonstrate is also evident during task engagement as a proxy for switching (See panels a-f in Extended Data Figure 5 in the current manuscript).

MD inactivation in the cueing sequence tasks (introduced in the current manuscript):

1. In the current manuscript, MD inactivation was never performed during the delay period. If it was, we expect it to have similar effects to the original task but since the focus on the neural activity was limited to evidence integration, we limited our manipulations to that period.
2. All manipulations were done in the steady-state behavior.

3. Under those conditions, we find that MD inactivation has minimal impact on the behavior when the cueing ambiguity is low (akin to what we find in the single cue version of the task). We interpret these findings as evidence of consistency in the role of the MD across these different tasks.

4. When the cueing ambiguity is increased, cue-specific MD inactivation has a detrimental effect on behavior. This is an original finding reported in this manuscript.

Supplemental Discussion:

Decision making is a topic of broad relevance, impacting several fields such as neuroscience, psychology, and economics[25,26]. Identifying the neurobiological substrates of how decisions are made, particularly when the factors driving them are ambiguous, is of great relevance. On one end, it can provide inspiration for designing artificial learning algorithms that can handle such ambiguity[27]. On another, it can be of clinical relevance given the high sensitivity to ambiguity observed in disorders such as schizophrenia[28,29] (See main text).

Our findings are broadly consistent with previous findings of frontal cortical activity patterns encoding task-relevant inputs (cues) and outputs (rules)[30–32], and MD thalamic responses being 'contextual' in nature[14,16]. These differences in encoding regimes may be related to differences in architectural features across these areas; although both structures are predominantly composed of excitatory glutamatergic neurons, the thalamus is devoid of local excitatory recurrent connections, a prominent feature of cortical organization[33]. The lack of local recurrence may actually be a design feature that allows these circuits to control cortical activity patterns that carry signals and noise independently. In addition, the nature of MD encoding of input uncertainty-related signals is also consistent with 'summary statistic' type responses seen in the Pulvinar of non-human primates performing a perceptual decision making (random dot motion) task[34,35].

Our data may speak to how fronto-thalamic circuits handle uncertainty in input statistics, categorizing it to low signals or high noise. It will be interesting to see how these circuits operate under different types of task-relevant uncertainty, such as those related to mapping (onto a rule) or outcome (probability of reward). This is particularly pertinent given the recent pioneering work on MD-PFC interactions in outcome uncertainty[31]. Such investigations are likely to yield important insights into how decision processes are organized, and how uncertainty at different hierarchical levels interact, perhaps through cortico-thalamic loops. In addition, it will likely be relevant for linking the computational abnormalities seen in schizophrenia to the underlying circuits to identify therapeutic targets in the process.

References

1. Bach, D. R., Hulme, O., Penny, W. D. & Dolan, R. J. The known unknowns: Neural representation of second-order uncertainty, and ambiguity. *Journal of Neuroscience* **31**, 4811–4820 (2011).

2. Fetsch, C. R., Pouget, A., Deangelis, G. C. & Angelaki, D. E. Neural correlates of reliability-based cue weighting during multisensory integration. *Nature Neuroscience* **15**, 146–154 (2012).

3. Bland, A. R. & Schaefer, A. Electrophysiological correlates of decision making under varying levels of uncertainty. *Brain Research* **1417**, 55–66 (2011).

4. O'Neill, M. & Schultz, W. Coding of reward risk by orbitofrontal neurons is mostly distinct from coding of reward value. *Neuron* **68**, 789–800 (2010).

5. Walker, E. Y., Cotton, R. J., Ma, W. J. & Tolias, A. S. A neural basis of probabilistic computation in visual cortex. *Nature Neuroscience* **23**, 122–129 (2020).

6. Yates, J. L., Park, I. M., Katz, L. N., Pillow, J. W. & Huk, A. C. Functional dissection of signal and noise in MT and LIP during decision-making. *Nature Neuroscience* **20**, 1285–1292 (2017).

7. Mante, V., Sussillo, D., Shenoy, K. v. & Newsome, W. T. Context-dependent computation by recurrent dynamics in prefrontal cortex. *Nature* **503**, 78–84 (2013).

8. Miller, E. K. & Buschman, T. J. Cortical circuits for the control of attention. *Current Opinion in Neurobiology* vol. 23 216–222 (2013).

9. Culbreth, A. J., Gold, J. M., Cools, R. & Barch, D. M. Impaired activation in cognitive control regions predicts reversal learning in schizophrenia. *Schizophrenia Bulletin* **42**, 484–493 (2016).

10. Wolff, M. & Vann, S. D. The cognitive thalamus as a gateway to mental representations. *Journal of Neuroscience* **39**, 3–14 (2019).

11. Parnaudeau, S., Bolkan, S. S. & Kellendonk, C. The Mediodorsal Thalamus: An Essential Partner of the Prefrontal Cortex for Cognition. *Biological Psychiatry* vol. 83 648–656 (2018).

12. Halassa, M. M. & Kastner, S. Thalamic functions in distributed cognitive control. *Nature Neuroscience* **20**, 1669–1679 (2017).

13. Bolkan, S. S. *et al.* Thalamic projections sustain prefrontal activity during working memory maintenance. *Nature neuroscience* **20**, 987–996 (2017).

14. Schmitt, L. I. *et al.* Thalamic amplification of cortical connectivity sustains attentional control. *Nature* **545**, 219–223 (2017).

15. Rikhye, R. v., Gilra, A. & Halassa, M. M. Thalamic regulation of switching between cortical representations enables cognitive flexibility. *Nature Neuroscience* **21**, 1753–1763 (2018).

16. Fresno, V., Parkes, S. L., Faugére, A. L., Coutureau, E. & Wolff, M. A thalamocortical circuit for updating action-outcome associations. *eLife* **8**, (2019).

17. Chakraborty, S., Kolling, N., Walton, M. E. & Mitchell, A. S. Critical role for the mediodorsal thalamus in permitting rapid reward-guided updating in stochastic reward environments. *eLife* **5**, (2016).

18. Grinband, J., Hirsch, J. & Ferrera, V. P. A neural representation of categorization uncertainty in the human brain. *Neuron* **49**, 757–763 (2006).

19. Kosciessa, J. Q., Lindenberger, U. & Garrett, D. D. Thalamocortical excitability modulation guides human perception under uncertainty. *Nature Communications* **12**, 1–15 (2021).

20. Giraldo-Chica, M., Rogers, B. P., Damon, S. M., Landman, B. A. & Woodward, N. D. Prefrontal-Thalamic Anatomical Connectivity and Executive Cognitive Function in Schizophrenia. *Biological Psychiatry* **83**, 509–517 (2018).

21. Bolkan, S. S. *et al.* Thalamic projections sustain prefrontal activity during working memory maintenance. *Nature Neuroscience* **20**, 987–996 (2017).

22. Hsiao, K. *et al.* A Thalamic Orphan Receptor Drives Variability in Short-Term Memory. *Cell* **183**, 522-536.e19 (2020).

23. Ferguson, B. R. & Gao, W. J. Thalamic Control of Cognition and Social Behavior Via Regulation of Gamma-Aminobutyric Acidergic Signaling and Excitation/Inhibition Balance in the Medial Prefrontal Cortex. *Biological Psychiatry* **83**, 657–669 (2018).

24. Rikhye, R. v., Gilra, A. & Halassa, M. M. Thalamic regulation of switching between cortical representations enables cognitive flexibility. *Nature Neuroscience* **21**, 1753–1763 (2018).

25. Gold, J. I. & Shadlen, M. N. The neural basis of decision making. *Annual Review of Neuroscience* vol. 30 535–574 (2007).

26. Tversky, A. & Kahneman, D. The framing of decisions and the psychology of choice. *Science* **211**, 453–458 (1981).

27. Masse, N. Y., Grant, G. D. & Freedman, D. J. Alleviating catastrophic forgetting using context dependent gating and synaptic stabilization. *Proceedings of the National Academy of Sciences of the United States of America* **115**, E104657–E104675 (2018).

28. Nassar, M., Waltz, J., Albrecht, M., Gold, J. & Frank, M. All or nothing belief updating in patients with schizophrenia reduces precision and flexibility of beliefs. *Brain* (2021) doi:10.1093/brain/awaa453.

29. Woodward, T. S., Mizrahi, R., Menon, M. & Christensen, B. K. Correspondences between theory of mind, jumping to conclusions, neuropsychological measures and the symptoms of schizophrenia. *Psychiatry Research* **170**, 119–123 (2009).

30. Wallis, J. D., Anderson, K. C. & Miller, E. K. Single neurons in prefrontal cortex encode abstract roles. *Nature* **411**, 953–956 (2001).

31. Alcaraz, F. *et al.* Thalamocortical and corticothalamic pathways differentially contribute to goal-directed behaviors in the rat. *eLife* **7**, (2018).

32. DeNicola, A. L., Park, M. Y., Crowe, D. A., MacDonald, A. W. & Chafee, M. v. Differential roles of mediodorsal nucleus of the thalamus and prefrontal cortex in decision-making and state representation in a cognitive control task measuring deficits in schizophrenia. *Journal of Neuroscience* **40**, 1650–1667 (2020).

33. Wimmer, R. D. *et al.* Thalamic control of sensory selection in divided attention. *Nature* **526**, 705–709 (2015).

34. Komura, Y., Nikkuni, A., Hirashima, N., Uetake, T. & Miyamoto, A. Responses of pulvinar neurons reflect a subject's confidence in visual categorization. *Nature Neuroscience* **16**, 749–755 (2013).

35. Jaramillo, J., Mejias, J. F. & Wang, X.-J. Engagement of Pulvino-cortical Feedforward and Feedback Pathways in Cognitive Computations. *Neuron* **101**, 321-336.e9 (2019).
