# Supplementary material for: Thalamic circuits for independent control of prefrontal signal and noise
Source: Nature. 2021 Oct 6;600(7887):100–4. doi: 10.1038/s41586-021-04056-3 (PMC8636261; doi:10.1038/s41586-021-04056-3)
Supplement: Supplementary file 2 — Reporting Summary [file 41586_2021_4056_MOESM2_ESM.pdf]

nature portfolio

Corresponding author(s): Michael Halassa

Last updated by author(s): Sep 11, 2021

Reporting Summary

Nature Portfolio wishes to improve the reproducibility of the work that we publish. This form provides structure for consistency and transparency in reporting. For further information on Nature Portfolio policies, see our Editorial Policies and the Editorial Policy Checklist.

Statistics

For all statistical analyses, confirm that the following items are present in the figure legend, table legend, main text, or Methods section.

n/a	Confirmed	
☐	☒	The exact sample size (*n*) for each experimental group/condition, given as a discrete number and unit of measurement
☐	☒	A statement on whether measurements were taken from distinct samples or whether the same sample was measured repeatedly
☐	☒	The statistical test(s) used AND whether they are one- or two-sided<br>*Only common tests should be described solely by name; describe more complex techniques in the Methods section.*
☐	☒	A description of all covariates tested
☐	☒	A description of any assumptions or corrections, such as tests of normality and adjustment for multiple comparisons
☐	☒	A full description of the statistical parameters including central tendency (e.g. means) or other basic estimates (e.g. regression coefficient) AND variation (e.g. standard deviation) or associated estimates of uncertainty (e.g. confidence intervals)
☐	☒	For null hypothesis testing, the test statistic (e.g. *F*, *t*, *r*) with confidence intervals, effect sizes, degrees of freedom and *P* value noted<br>*Give P values as exact values whenever suitable.*
☒	☐	For Bayesian analysis, information on the choice of priors and Markov chain Monte Carlo settings
☒	☐	For hierarchical and complex designs, identification of the appropriate level for tests and full reporting of outcomes
☐	☒	Estimates of effect sizes (e.g. Cohen's *d*, Pearson's *r*), indicating how they were calculated

Our web collection on statistics for biologists contains articles on many of the points above.

Software and code

Policy information about availability of computer code

Data collection	Confocal images, with z-planes, of tissue sections were obtained on a Zeiss LSM710 confocal microscope using ZEN2012 image acquisition software. Z-planes were reconstructed and rendered into 3D images for quantification using IMARIS image analysis software (v9.3.2). Extracellular spikes were recorded with Cheetah Data Acquisition System (v6.4) on a Digital Lynx SX system from Neuralynx. In order to record the timestamp and identity of task events (trial initiation, cue presentation correct vs incorrect choice, etc) from each behavioral session, the Arduino micro-controller used to control the behavioral apparatus was connected to a PC using a serial port, and the data were acquired into a data matrix generated through MATLAB.<br>The following softwares are used in this study: ImageJ (1.51w), SolidWorks (v23).
Data analysis	All computer code used for analysis in this study was implemented in MATLAB computing software (MathWorks, R2020b). Some statsitical analyses was performed in Graphpad Prism software (v8.0) instead. Model simulation was implemented in Python (v2.7). Code will be made freely available to any party upon request. Requests should be directed to the corresponding author.

For manuscripts utilizing custom algorithms or software that are central to the research but not yet described in published literature, software must be made available to editors and reviewers. We strongly encourage code deposition in a community repository (e.g. GitHub). See the Nature Portfolio guidelines for submitting code & software for further information.

Data

Policy information about availability of data

All manuscripts must include a data availability statement. This statement should provide the following information, where applicable:

- Accession codes, unique identifiers, or web links for publicly available datasets
- A description of any restrictions on data availability
- For clinical datasets or third party data, please ensure that the statement adheres to our policy

We include the following statement of data availability in the methods section: "The data that support the findings of this study are available from the corresponding author upon reasonable request."

Field-specific reporting

Please select the one below that is the best fit for your research. If you are not sure, read the appropriate sections before making your selection.

☒ Life sciences ☐ Behavioural & social sciences ☐ Ecological, evolutionary & environmental sciences

For a reference copy of the document with all sections, see nature.com/documents/nr-reporting-summary-flat.pdf

Life sciences study design

All studies must disclose on these points even when the disclosure is negative.

Sample size	We include the following statement of data availability in the methods section: "The data that support the findings of this study are available from the corresponding author upon reasonable request."
Data exclusions	For clusters corresponding to the spikes of individual recorded neurons, only well isolated clusters with biologically plausible waveforms were selected for further analysis as described in the methods. In addition, to reduce noise, neurons with low base rate (<0.5Hz) were excluded from further analysis.<br><br>For all anatomical, electrophysiological, behavioral analyzes, animals where virus injection sites missed the target location were discarded from further analysis. Reported numbers are only for included animals.
Replication	Each figure legend contains a description of the number of animals (biological replicates) as well as the technical replicates in the form of number of sessions (behavior) or number of cells (anatomy, electrophysiology) or number of sections a data set was acquired from.<br><br>Regarding replication of experimental findings in previous work, we include the following statement in the results section: "Previous work indicated that driving the MD has two distinct effects on PL neural activity: amplification of local functional connectivity (ref 14) and suppression of spike rates (ref 21). We replicated these effects in a new cohort of animals (Fig. S1, Fig. 1)"
Randomization	Across experiments, mice were randomly selected for behavioral training and anatomical studies. For behavioral tasks all mice trained to criteria were included in testing, and all mice trained to criteria were included in testing. For optogenetic studies and physiological recording, mice were randomly selected from the overall cohort for inclusion in each type of manipulation/recording.
Blinding	For all optogenetic experiments (Fig. 3-5, Fig. Extended Data Fig. 9 and 14), optogenetic trials were randomly interleaved among other trial types and investigators were blind to trial type, longitudinal comparisons were then used within individuals between trial types. This is true except for experiments where the role of MD in task engagement was evaluated (Extended Data Fig. 7, first 30 trials in the sessions are optogenetic), or to the optotagging experiments (Fig. 4a, optogenetic trials at the end, after the behavior session).<br><br>Blinding to group allocation is not applicable to this study - there are no treatment groups. Animal genotype are known to the investigators.

Reporting for specific materials, systems and methods

We require information from authors about some types of materials, experimental systems and methods used in many studies. Here, indicate whether each material, system or method listed is relevant to your study. If you are not sure if a list item applies to your research, read the appropriate section before selecting a response.

Materials & experimental systems

n/a	Involved in the study	
☐	☒	Antibodies
☒	☐	Eukaryotic cell lines
☒	☐	Palaeontology and archaeology
☐	☒	Animals and other organisms
☒	☐	Human research participants
☒	☐	Clinical data
☒	☐	Dual use research of concern

Methods

n/a	Involved in the study	
☒	☐	ChIP-seq
☒	☐	Flow cytometry
☒	☐	MRI-based neuroimaging

Antibodies

Antibodies used

Antibodies used in this study were described the methods section of the text: chicken anti-GFP antibody (1:1000, Aves Labs, GFP1011); Alexa Fluor 488 goat anti-chicken secondary antibody (1:500, Thermofisher, A32931); rabbit anti-PV (1:1000, Swant, PV-27) primary antibody; rabbit anti-VIP (1:200, Immunostar, 20077), rabbit anti-Grik4 (1:100, Alomone labs, AGC-041) primary antibodies; Alexa Fluor 647 donkey anti-rabbit secondary antibody (1:200, Thermofisher, A31573).

Validation

All antibodies were purchased from commercial vendors who have performed initial validation. As a secondary measure we only used antibodies that have been used in published studies. Antibodies used in this study were described the methods section of the text:
chicken anti-GFP primary antibody (1:1000, Aves Labs, GFP1011; Reference: Efrain A. Ribeiro, Alexander R. Nectow, Lisa E. Pomeranz, Mats I. Ekstrand, Ja Wook Koo, Eric J. Nestler (2019), 'Viral labeling of neurons synaptically connected to nucleus accumbens somatostatin interneurons.' Plos One. 10.1371/journal.pone.0213476.)
rabbit anti-PV primary antibody (1:1000, Swant, PV-27; Reference: Mukherjee A, Carvalho F, Eliez S, Caroni P. Long-Lasting Rescue of Network and Cognitive Dysfunction in a Genetic Schizophrenia Model. Cell. 2019 Sep 5;178(6):1387-1402.e14. doi: 10.1016/j.cell.2019.07.023. Epub 2019 Aug 29. PMID: 31474363.)
rabbit anti-VIP primary antibody (1:200, Immunostar, 20077; Reference: Alamilla J, Ramiro-Cortés Y, Mejía-López A, Chavez JL, Rivera DO, Felipe V, Aguilar-Roblero R. Altered Light Sensitivity of Circadian Clock in Shank3+/- Mouse. Front Neurosci. 2021 Feb 18;15:604165. doi: 10.3389/fnins.2021.604165. PMID: 33679297; PMCID: PMC7930753.)
rabbit anti-Grik4 (1:100, Alomone labs, AGC-041; Reference: Bhandage AK, Jin Z, Hellgren C, Korol SV, Nowak K, Williamsson L, Sundström-Poromaa I, Birnir B. AMPA, NMDA and kainate glutamate receptor subunits are expressed in human peripheral blood mononuclear cells (PBMCs) where the expression of GluK4 is altered by pregnancy and GluN2D by depression in pregnant women. J Neuroimmunol. 2017 Apr 15;305:51-58. doi: 10.1016/j.jneuroim.2017.01.013. Epub 2017 Jan 21. PMID: 28284346.)
Alexa Fluor 488 goat anti-chicken secondary antibody (1:500, Thermofisher, A32931; Reference: Hanson E, Swanson J, Arenkiel BR. GABAergic Input From the Basal Forebrain Promotes the Survival of Adult-Born Neurons in the Mouse Olfactory Bulb. Front Neural Circuits. 2020 Apr 23;14:17. doi: 10.3389/fncir.2020.00017. PMID: 32390805; PMCID: PMC7190813.);
Alexa Fluor 647 donkey anti-rabbit secondary antibody (1:200, Thermofisher, A31573, Reference: Hellström Erkenstam N, Smith PL, Fleiss B, Nair S, Svedin P, Wang W, Boström M, Gressens P, Hagberg H, Brown KL, Sävman K, Mallard C. Temporal Characterization of Microglia/Macrophage Phenotypes in a Mouse Model of Neonatal Hypoxic-Ischemic Brain Injury. Front Cell Neurosci. 2016 Dec 15;10:286. doi: 10.3389/fncel.2016.00286. PMID: 28018179; PMCID: PMC5156678.).

Animals and other organisms

Policy information about studies involving animals; ARRIVE guidelines recommended for reporting animal research

Laboratory animals

A total of 94 mice were used in this study. Adult C57Bl/6 (WT) mice, of both sexes, aged 8-12 weeks old were purchased from Taconic Biosciences. GRIK4-cre, PV-cre, VIP-cre and SST-cre mice, of both sexes and ages between 8-12weeks, were obtained from the Jackson laboratories. D2-cre mice (GENSAT, line ER44), of both sexes between ages 8 to 12 weeks were a kind gift from Dr. Myriam Heiman, MIT. Cre mice were backcrossed to C57Bl/6 mice for at least six generations. All mice were kept in rooms with controlled temperature and ventilation (20-22 degrees Celsius; 40-60% humidity) on a constant 12h light-dark cycle. Animals were group housed with ad libidum access to food and water.

Wild animals

The study did not involve any wild animals.

Field-collected samples

The study did not involve samples collected from the field.

Ethics oversight

All animal experiments were performed according to the guidelines of the US National Institutes of Health and the Institutional Animal Care and Use Committee at the Massachusetts Institute of Technology.

Note that full information on the approval of the study protocol must also be provided in the manuscript.
